# Hirota, Fay and geometry

**DOI:** 10.1007/s11005-025-01978-1

**Published:** 2025-08-21

**Authors:** B. Eynard, S. Oukassi

**Affiliations:** 1 CNRS, CEA, Institut de Physique Théorique, Université Paris-Saclay, 91191 Gif-sur-Yvette, France; 2 CRM, Centre de Recherches Mathématiques de Montréal, Université de Montréal, Montreal, QC Canada

**Keywords:** Hirota equations, Fay identities, Topological recursion, Compact Riemann surfaces, 37K10, 14H70, 14H42

## Abstract

This is a review of the relationship between Fay identities and Hirota equations in integrable systems, reformulated in a geometric language compatible with recent Topological Recursion formalism. We write Hirota equations as trans-series and Fay identities as spinor functional relations. We also recall several constructions of how some solutions to Fay/Hirota equations can be built from Riemann surface geometry.

## Introduction: Tau functions and Hirota

Tau functions are central to the theory of integrable systems; we shall refer to [6, 7, 49] for further references on integrable systems and Tau functions.

Tau functions are usually presented as functions of an infinite set of variables and are solutions of an infinite set of nonlinear partial differential equations.

The most famous are “KdV”, “KP” and “Toda” Tau functions.

The set of equations that they satisfy is called “Hirota equations” and can be rephrased as Fay identities.

In the form of Fay identities, the Hirota equations become equations for germs of functions of a finite number of complex variables.

It is thus natural to look for solutions of Hirota/Fay equations where the complex variables are actually local coordinates on a Riemann surface and thus associate Tau functions to Riemann surfaces.

We recall here some Riemann surface theoretic solutions of Hirota/Fay identities.

**Outline:**

Section 1 is a brief introduction to Tau functions, Sato vectors and Hirota equations in the formalism of Hirota, Sato, Segal–Wilson, and others.

Section 2: in the usual Hirota equations, Tau functions are formal series and are multiplied by exponentials of negative powers, and Hirota equations involve a residue, i.e., an integral with a differential form $d\xi$. It is thus natural to reformulate Tau functions including exponentials and differentials, as spinor trans-series.

Section 3: we recall how Hirota equations lead to Fay identities. In the sense of formal series, this would be equivalent, but Fay identities can be extended beyond formal series.

Section 4: Fay identities are equations relating germs of analytic functions, and it is thus natural to view them as equations of analytic functions on a Riemann surface. In other words, one can look for solutions of Fay identities in the world of Riemann surfaces. Section 4 is a brief summary of the geometry of Riemann surfaces.

Section 5: we show one classical solution of Fay identities on Riemann surfaces, using Theta functions; this is the famous “reconstruction formula” for integrable systems.

Section 6: we show another solution of Fay identities on a moduli space of spectral curves (Riemann surface with an immersion into a cotangent space), as an asymptotic formula using “Topological Recursion”.

Section 7: we mention other solutions, and particularly the “matrix integrals”.

Section 8: we gather a number of concluding remarks.

### Tau functions as graded formal series

A now standard formulation of Tau functions was introduced by Sato, Miwa, Jimbo and the Japanese school [57–60, 74, 77], and in another point of view by Segal and Wilson [76]. Here we stay closer to the formulation of Sato, which we present here as a *formal* Tau function that depends on an infinite set of graded coordinates called “times”

1.1 $$\begin{aligned} \textbf{t}= (t_1,t_2,t_3,\dots ) \qquad , \quad \deg t_k = k. \end{aligned}$$

In the formulation of Sato and Segal–Wilson, the times are coordinates on an Abelian group $\Gamma$ of transformations acting on an infinite-dimensional Grassmannian, but we shall not need these notions here.

The Tau function (we denote it $\hat{{\mathcal {T}}}$ to distinguish from the slightly renormalized version ${\mathcal {T}}$ that we shall consider below)

1.2 $$\begin{aligned} \hat{{\mathcal {T}}}(\textbf{t}) \in {\mathbb {C}}[[\Gamma ]] = {\mathbb {C}}[[t_1,t_2,t_3,\dots ]] \end{aligned}$$

is a formal graded series, whose truncation to any degree is a polynomial in the times, which obeys an infinite set of partial differential or functional equations that we shall review below.

#### Definition 1.1

(*Sato’s vector*) Let $\xi \in {\mathbb {C}}$ define the following vector (called after Sato):

1.3 $$\begin{aligned} {[}\xi ] := (\xi ,\xi ^{2},\xi ^{3},\xi ^4,\dots ) \in \Gamma . \end{aligned}$$

In the filtration, $\xi$ has degree 1.

#### Remark 1.1

Here we slightly change the usual Sato’s normalization of times, by multiplying by *k*. Our times are

1.4 $$\begin{aligned} t_k = k \times \text {Sato's time }t_k. \end{aligned}$$

This multiplication by *k* is useful for the comparison with TR (Topological Recursion) formalism and is necessary to match the times with the canonical Darboux coordinates in the space of generalized homology cycles [36].

#### Definition 1.2

(*Divisors*) A divisor *D* is a set of distinct points $z_1,\dots ,z_\ell$ of the complex plane $z_i\in {\mathbb {C}}$, with some respective weights $\alpha _1,\dots ,\alpha _\ell$, where $\alpha _i\in {\mathbb {C}}$. Weights are also called “charges”. It is often denoted (this is merely a notation) as a weighted sum of points:

1.5 $$\begin{aligned} D := \sum _{i=1}^\ell \alpha _i . z_i \ . \end{aligned}$$

The sum of weights, the total charge, is called the degree of the divisor:

1.6 $$\begin{aligned} \deg D := \sum _{i=1}^\ell \alpha _i. \end{aligned}$$

The support of the divisor is the set of points with nonzero weight:

1.7 $$\begin{aligned} \operatorname {supp}D := \{z_i \ | \ \alpha _i\ne 0\}. \end{aligned}$$

The set of divisors is a module by addition and scalar multiplication of the weights.

A divisor *D* is said **neutral** if $\deg D=0$. A divisor is said **integer** if weights are integers, and is **unitary** if weights are $\pm 1$. A divisor is said **positive** or **bosonic** (resp. **negative** or **fermionic**) if all weights are positive (resp. negative). A divisor is said **supsersymmetric** if it is of degree 0 and all weights are $\pm 1$. A supersymmetric divisor must have an even number of points, half of them have weight $+1$ (bosons) and half of them have weight $-1$ (fermions).

#### Definition 1.3

(*Sato’s vector for divisors*) We associate a Sato vector to a divisor $D=\sum _i \alpha _i.z_i$, equal to the weighted linear combination of Sato vectors of its points:

1.8 $$\begin{aligned} {[}D] = \sum _{i=1}^\ell \alpha _i [z_i] = (D_1,D_2,D_3,\dots ) \in \Gamma \quad \text {where}\quad D_k = \sum _{i=1}^\ell \alpha _i z_i^{k}. \end{aligned}$$

It is convenient to think of *D* as a diagonal matrix with eigenvalues $z_i$ and multiplicities $\alpha _i$ ($\alpha _i<0$ means “super-matrix” with “fermionic” eigenvalues). With the diagonal matrix notation we have:

1.9 $$\begin{aligned} D_k = {\operatorname {Tr}}D^{k}. \end{aligned}$$

#### Remark 1.2

(*Screening: Divisors of degree 0*) Remark that if $z=0$, then the Sato vector $[z] = (0,0,0,\dots )$ is the null vector in $\Gamma$. Therefore, if a divisor $D=\sum _{i=1}^\ell \alpha _i [z_i]$ has a nonzero degree, $\deg D\ne 0$, we can always supplement it by appending to it the point

$$z_0=0, \qquad \alpha _0=-\deg D.$$

We get a new divisor whose degree is 0

1.10 $$\begin{aligned} \tilde{D}=D+\alpha _0.0, \quad \deg \tilde{D}=0, \end{aligned}$$

and such that

1.11 $$\begin{aligned} \forall \ k>0 \ \qquad \tilde{D}_k=D_k={\operatorname {Tr}}\tilde{D}^k ={\operatorname {Tr}}D^k, \end{aligned}$$

i.e., as a vector in $\Gamma$

1.12 $$\begin{aligned} {[}\tilde{D}] = (D_1,D_2,D_3,\dots ) = [D]. \end{aligned}$$

In other words, it is not restrictive to require that a divisor has degree 0; it can be completed by the point 0 with the complementary weight. This is called “screening charge”.

### Hirota equation

Hirota equation is a set of nonlinear PDEs satisfied by $\hat{{\mathcal {T}}}$, and gathered into a bilinear relation that generates all of them:

#### Definition 1.4

(*Hirota Equation*) $\hat{{\mathcal {T}}}(\vec {t})$ is said to satisfy Hirota equation iff for all $\ell$ a positive integer, and all $\mu =(\mu _1,\mu _2,\dots ,\mu _\ell )\in \mathbb N^\ell$ a $\ell$-upple, we have

1.13 $$\begin{aligned} \mathop {{\textrm{Res}}}_{\xi \rightarrow 0} \,[u_1^{\mu _1} u_2^{\mu _2}\dots u_\ell ^{\mu _\ell }] \left( \hat{{\mathcal {T}}}(\vec {t} + \vec {u}+ [\xi ]) \hat{{\mathcal {T}}}(\vec {t}-\vec {u} - [\xi ]) e^{- 2\sum _{k=1}^{\ell } \frac{u_k}{k}\xi ^{-k} } \right) \frac{d\xi }{\xi ^2} = 0. \end{aligned}$$

The notation $[u^\mu ](.)= [u_1^{\mu _1} u_2^{\mu _2}\dots u_\ell ^{\mu _\ell }] \left( . \right)$ means that we first expand the parenthesis as a power series of the $u_k$s and extract the coefficient of the monomial $u_1^{\mu _1} u_2^{\mu _2}\dots u_\ell ^{\mu _\ell }$, and then, we compute the residue at $\xi \rightarrow 0$.

This is well defined, because the exponential is equal to its Taylor series in the $u_k$s (absolutely convergent moreover), and since $\hat{{\mathcal {T}}}$ is a graded formal series, for a given $\ell$-upple $\mu$, there is at most a finite number of terms contributing to the monomial $[u^\mu ]$. The expression is then a polynomial of $\xi ^{-1}$, times a formal power series of $\xi$, and therefore, its residue is well defined (the coefficient of $\xi ^{-1}$).

In fact, **Hirota** introduced it, because he had realized that this equation was common to almost all integrable systems known at that time:

#### Theorem 1.1

(Hirota) The Tau functions of the KdV, KP, Toda hierarchies satisfy the Hirota equation.

For the proof, we refer to the literature [6, 7, 49–51].

#### Turning Hirota to PDEs

Let us review [6, 7, 49] how Hirota equation yields an infinite set of nonlinear PDEs.

We have, in the sense of formal series in ${\mathbb {C}}[[\xi ,u_1,u_2,\dots ]]$, the Taylor formula:

1.14 $$\begin{aligned} \hat{{\mathcal {T}}}(\vec {t} + \vec {u}+ [\xi ]) = e^{\sum _{k=1}^\infty (u_k+\xi ^{k})\frac{\partial }{\partial t_k} }\hat{{\mathcal {T}}}(\vec {t}). \end{aligned}$$

Since Hirota equation is bilinear and involves two Tau functions, some derivatives will act onto the 1st Tau function, and some onto the 2nd. We shall write one Tau function at the left, the other at the right, and define two operators $\overset{\leftarrow }{\frac{\partial }{\partial t_k}}$ and $\overset{\rightarrow }{\frac{\partial }{\partial t_k}}$. We also introduce the differential bi-operator :

1.15 $$\begin{aligned} \mathcal {D}_k := \overset{\leftarrow }{\frac{\partial }{\partial t_k}} - \overset{\rightarrow }{\frac{\partial }{\partial t_k}}. \end{aligned}$$

This means that in ${\mathbb {C}}[[\xi ,u_1,u_2,\dots ]]$

1.16 $$\begin{aligned} \hat{{\mathcal {T}}}(\vec {t} + \vec {u}+ [\xi ]) \hat{{\mathcal {T}}}(\vec {t} - \vec {u}- [\xi ])= &   \hat{{\mathcal {T}}}(\vec {t}) e^{\sum _k (u_k+\xi ^{k})\overset{\leftarrow }{\frac{\partial }{\partial t_k}} } e^{-\sum _k (u_k+\xi ^{k})\overset{\rightarrow }{\frac{\partial }{\partial t_k}} }\hat{{\mathcal {T}}}(\vec {t}) \\= &   \hat{{\mathcal {T}}}(\vec {t}) e^{\sum _k (u_k+\xi ^{k}) D_k }\hat{{\mathcal {T}}}(\vec {t}). \end{aligned}$$

Hirota equations can then be written as follows:

##### Theorem 1.2

(Hirota as PDEs) For all $\mu =(\mu _1,\dots ,\mu _\ell )$

1.17 $$\begin{aligned} \hat{{\mathcal {T}}}(\vec {t}) \ \mathcal {D}_\mu \ \hat{{\mathcal {T}}}(\vec {t}) = 0, \end{aligned}$$

where $\mathcal {D}_\mu$ is a differential bi-operator defined as

1.18 $$\begin{aligned} \mathcal {D}_\mu = \left( \mathop {{\textrm{Res}}}_{\xi \rightarrow 0}\, \frac{d\xi }{\xi ^2} \ e^{\sum _k \xi ^{k}D_k} \ \prod _{j=1}^\ell \frac{\left( \mathcal {D}_j-\frac{2}{j}\xi ^{-j}\right) ^{\mu _j}}{\mu _j!}\right) . \end{aligned}$$

$\mathcal {D}_\mu$ is a polynomial of $\mathcal {D}_1,\mathcal {D}_2,\mathcal {D}_3,\dots$ of graded homogeneous degree $1+\sum _{i=1}^\ell i\mu _i$. More explicitly:

1.19 $$\begin{aligned}  &   \mathcal {D}_\mu = \sum _{\sum _i i m_i + \sum _j j\alpha _j = 1+\sum _j j \mu _j} \ \ \prod _{j=1}^\ell \left( \frac{-2}{j}\right) ^{\mu _j-\alpha _j} \ \frac{\mathcal {D}_j^{\alpha _j}}{\alpha _j! (\mu _j-\alpha _j)!} \ \prod _{i\ge 1} \frac{\mathcal {D}_i^{m_i}}{m_i!}\nonumber \\ \end{aligned}$$

##### Remark 1.3

Remark that $\hat{{\mathcal {T}}}(\vec {t}) \ \mathcal {D}_\mu \ \hat{{\mathcal {T}}}(\vec {t})$ is symmetric in the exchange of the ${\mathcal {T}}$ at the left and at the right. This implies that $\hat{{\mathcal {T}}}(\vec {t}) \ \mathcal {D}_\mu \ \hat{{\mathcal {T}}}(\vec {t})$ is unchanged if we change each $\mathcal {D}_k\rightarrow -\mathcal {D}_k$. In other words, we may keep in $\mathcal {D}_\mu$ only the terms that are even in the change $\mathcal {D}_k\rightarrow -\mathcal {D}_k$. Any odd polynomial of $\mathcal {D}_1,\mathcal {D}_2,\mathcal {D}_3,\dots$ will trivially give 0 in the equation $\hat{{\mathcal {T}}}(\vec {t}) \ \mathcal {D}_\mu \ \hat{{\mathcal {T}}}(\vec {t})$.

##### Example 1.1

1.20 $$\begin{aligned} \mathcal {D}_{(0,0,0,\dots )} = \mathcal {D}_1, \quad \mathcal {D}_{(1,0,0,\dots )} = -2\mathcal {D}_2,\quad \mathcal {D}_{(0,1,0,\dots )} = -\frac{1}{6} \mathcal {D}_1^3-\mathcal {D}_3 \end{aligned}$$

1.21 $$\begin{aligned} \mathcal {D}_{(2,0,0,\dots )} = -\frac{1}{6} \mathcal {D}_1^3+2\mathcal {D}_3,\quad \mathcal {D}_{(0,0,1,0,\dots )} =-\frac{\mathcal {D}_{1}^{4}}{36} - \frac{\mathcal {D}_{1}^{2} \mathcal {D}_{2}}{3} + \frac{\mathcal {D}_{1} \mathcal {D}_{3}}{3} - \frac{\mathcal {D}_{2}^{2}}{3} - \frac{2 \mathcal {D}_{4}}{3} \end{aligned}$$

1.22 $$\begin{aligned} \mathcal {D}_{(2,1,0,\dots )} = -\frac{\mathcal {D}_{1}^{5}}{60} + \frac{\mathcal {D}_{1}^{2} \mathcal {D}_{3}}{2} - 2 \mathcal {D}_{5}. \end{aligned}$$

For example, $\hat{{\mathcal {T}}} \mathcal {D}_{(0,0,1,0,\dots )}\hat{{\mathcal {T}}} = 0$ implies the following differential equation

1.23 $$\begin{aligned} 0= &   \hat{{\mathcal {T}}} \ \partial _{t_2}^2 \hat{{\mathcal {T}}} - \partial _{t_2}\hat{{\mathcal {T}}} \ \partial _{t_2}\hat{{\mathcal {T}}} +\frac{1}{12}\Big (\hat{{\mathcal {T}}} \ \partial {t_1}^4 \hat{{\mathcal {T}}} - 4 \partial _{t_1}\hat{{\mathcal {T}}} \ \partial {t_1}^3 \hat{{\mathcal {T}}} + 3 \partial _{t_1}^2\hat{{\mathcal {T}}} \ \partial {t_1}^2 \hat{{\mathcal {T}}} \Big ) \\  &   - \hat{{\mathcal {T}}} \ \partial {t_1}\partial _{t_3} \hat{{\mathcal {T}}} + \partial {t_1} \hat{{\mathcal {T}}} \ \partial _{t_3} \hat{{\mathcal {T}}}. \end{aligned}$$

If we write $\hat{{\mathcal {T}}} = e^{\hat{F}}$, we get the KP equation

1.24 $$\begin{aligned} 0= &   \partial _{t_2}^2 \hat{F} +\frac{1}{12}\Big (\partial _{t_1}^4 \hat{F}+6 (\partial _{t_1}\hat{F})^2 \Big ) - \partial _{t_3}\partial _{t_1} \hat{F}. \end{aligned}$$

### Insertion operator

#### Definition 1.5

(*Insertion operator (perturbative)*) We define

1.25 $$\begin{aligned} \Delta _\xi := d\xi \sum _{k=1}^\infty k \xi ^{k-1} \frac{\partial }{\partial t_k} = d\left( \sum _{k=1}^\infty \xi ^{k} \frac{\partial }{\partial t_k} \right) . \end{aligned}$$

$\Delta$ is a 1-form valued differential operator. As defined here, it acts on the space ${\mathbb {C}}[[\Gamma ]]$ of graded formal series of the times and results in a formal series of $\xi$, times $d\xi$:

1.26 $$\begin{aligned} \Delta : {\mathbb {C}}[[t_1,t_2,\dots ]] \rightarrow {\mathbb {C}}[[t_1,t_2,\dots ]][[\xi ]] d\xi . \end{aligned}$$

#### Lemma 1.1

(Insertion operator and shifted times) For any $f(\vec {t})\in {\mathbb {C}}[[\Gamma ]]$, we have (in the sense of formal graded series)

1.27 $$\begin{aligned} f(\vec {t}+\alpha [\xi ]) = e^{\alpha \int _0^\xi \Delta } f(\vec {t}), \end{aligned}$$

1.28 $$\begin{aligned} \Delta _\xi f(\vec {t}) = \lim _{\alpha \rightarrow 0}\left( \frac{1}{\alpha }d\xi \ \frac{d}{d\xi }f(\vec {t}+\alpha [\xi ])\right) . \end{aligned}$$

1.29 $$\begin{aligned} f(\vec {t}+\alpha [\xi ]-\alpha [\tilde{\xi }]) = e^{\alpha \int _{\tilde{\xi }}^\xi \Delta } f(\vec {t}), \end{aligned}$$

1.30 $$\begin{aligned} \Delta _\xi f(\vec {t}) = \lim _{\alpha \rightarrow 0}\left( \frac{1}{\alpha }d\xi \ \frac{d}{d\xi }f(\vec {t}+\alpha [\xi ]-\alpha [\tilde{\xi }])\right) . \end{aligned}$$

#### Proof

From Definition 1.5, we can write:

1.31 $$\begin{aligned} e^{\alpha \int _{\tilde{\xi }}^\xi \Delta } f(\vec {t})= &   e^{\sum _{k=1}^{\infty }\alpha \left( \xi ^k-\tilde{\xi }^k\right) \frac{\partial }{\partial t_k} } \ f(t_1,t_2,\dots ) \\= &   f(\vec {t} + \alpha [\xi ]-\alpha [\tilde{\xi }]). \end{aligned}$$

The exponentiel term, viewed as an element of $\mathbb {C}[[\xi ,\tilde{\xi }]]$, can be written as:

1.32 $$\begin{aligned} e^{\sum _{k=1}^{\infty }\alpha \left( \xi ^k-\tilde{\xi }^k\right) \frac{\partial }{\partial t_k} }f(\vec {t})=\sum _{l=0}^{\infty } \frac{\alpha ^l}{l!}\left( \sum _{k=1}^{\infty } (\xi ^k-\tilde{\xi }^k) \frac{\partial }{\partial t_k} \right) ^l f(\vec {t}), \end{aligned}$$

1.33 $$\begin{aligned} \frac{d}{d\xi }f(\vec {t}+\alpha [\xi ]-\alpha [\tilde{\xi }])=\sum _{l=1}^{\infty } \frac{\alpha ^l}{(l-1)!}\left( \sum _{k=1}^{\infty } (\xi ^k-\tilde{\xi }^k) \frac{\partial }{\partial t_k} \right) ^{l-1} \left( \sum _{k=1}^{\infty } k \xi ^{k-1} \frac{\partial }{\partial t_k}\right) f(\vec {t}). \end{aligned}$$

Hence, we have

1.34 $$\begin{aligned} \lim _{\alpha \rightarrow 0}\left( \frac{1}{\alpha }d\xi \ \frac{d}{d\xi }f(\vec {t}+\alpha [\xi ]-\alpha [\tilde{\xi }])\right) =\sum _{k=1}^{\infty } k \xi ^{k-1} \frac{\partial }{\partial t_k}f(\vec {t})=\Delta _\xi f(\vec {t}). \end{aligned}$$

Moreover, Eq. (1.28) is obtained from Eq. (1.30) by taking the limit $\tilde{\xi }\rightarrow 0$, as well as Eq. (1.27). $\square$

#### Remark 1.4

Equation (1.25) is the Taylor series expansion of Eq. (1.30) or (1.28). From now on, we shall use Eq. (1.30) as the definition of the insertion operator $\Delta$ instead of Eq. (1.25), because it is now defined not only as a germ but as a genuine analytic function of $\xi$, and it can act on functions of complex variables rather than formal series of times.

#### Definition 1.6

(*Potential*) Let

1.35 $$\begin{aligned} V_{\vec {t}}(\xi ) :=\sum _{k=1}^\infty \frac{t_k}{k} \xi ^{-k} \in {\mathbb {C}}[[t_1,t_2,\dots ]][[\xi ^{-1}]]. \end{aligned}$$

We have

1.36 $$\begin{aligned} dV_{\vec {t}}(\xi ) = -\sum _{k=1}^\infty t_k \xi ^{-k-1}d\xi , \end{aligned}$$

1.37 $$\begin{aligned} \Delta _{\xi _2}dV_{\vec {t}}(\xi _1) = - \frac{d\xi _1 \otimes d\xi _2}{(\xi _1-\xi _2)^2}. \end{aligned}$$

#### Remark 1.5

If times grow like $|t_k|=O(R^{k})$ at large *k* for some $R>0$, then $V_{\vec {t}}(\xi )$ is analytic in a disk around $\infty$ of radius $|\xi |\ge R$.

#### Remark 1.6

Although $\frac{d\xi _1 \otimes d\xi _2}{(\xi _1-\xi _2)^2}$ is a well-defined meromorphic bilinear differential form, known as the “fundamental second kind differential” or sometimes “Bergman kernel” of the sphere ${\mathbb {C}}P^1$, it is not a formal series in neither $\xi _1^{-1}$ nor $\xi _2^{-1}$. However, its Taylor expansion at large $\xi _1$ for fixed $\xi _2$, or vice versa, are Laurent formal series, and will be used as such, as soon as one precises which variable is kept fixed and which is considered as formal expansion variable. This definition is actually not used in the derivations below and is just for bookkeeping for matching the Topological Recursion formalism.

#### Definition 1.7

(*Correlators*) The following quantities are called the “correlators”:

1.38 $$\begin{aligned} \hat{W}_0(\vec {t}) := \ln \hat{{\mathcal {T}}}(\vec {t}), \end{aligned}$$

1.39 $$\begin{aligned} W_1(\vec {t};\xi ) := \Delta _\xi \ln \hat{{\mathcal {T}}}(\vec {t}) \ +\sum _{k\ge 1} t_k \xi ^{-k-1}d\xi = \Delta _\xi \ln \hat{{\mathcal {T}}}(\vec {t}) \ -dV_{\vec {t}}(\xi ), \end{aligned}$$

1.40 $$\begin{aligned} W_2(\vec {t};\xi _1,\xi _2) := \Delta _{\xi _1}\otimes \Delta _{\xi _2} \ln \hat{{\mathcal {T}}}(\vec {t}) \ + \frac{d\xi _1 \otimes d\xi _2}{(\xi _1-\xi _2)^2}, \end{aligned}$$

and for $$n\ge 3$$

1.41 $$\begin{aligned} W_n(\vec {t};\xi _1,\xi _2,\dots ,\xi _n) := \Delta _{\xi _1}\otimes \Delta _{\xi _2}\otimes \dots \otimes \Delta _{\xi _n} \ln \hat{{\mathcal {T}}}(\vec {t}) . \end{aligned}$$

#### Lemma 1.2

For $$k>0$$

1.42 $$\begin{aligned} t_k = \mathop {{\textrm{Res}}}_{\xi \rightarrow 0} \,\xi ^{k} \ W_1(\vec {t};\xi ), \end{aligned}$$

1.43 $$\begin{aligned} \frac{\partial }{\partial t_k}\ln \hat{{\mathcal {T}}}(\vec {t}) = \frac{1}{k} \mathop {{\textrm{Res}}}_{\xi \rightarrow 0} \,\xi ^{-k} \ W_1(\vec {t};\xi ). \end{aligned}$$

For $$k_1,k_2,\dots ,k_n>0$$

1.44 $$\begin{aligned} \frac{\partial }{\partial t_{k_1}}\dots \frac{\partial }{\partial t_{k_n}}\ln \hat{{\mathcal {T}}}(\vec {t}) = \frac{1}{k_1\dots k_n} \mathop {{\textrm{Res}}}_{\xi _1\rightarrow 0} \,\dots \mathop {{\textrm{Res}}}_{\xi _n\rightarrow 0}\, \xi _1^{-k_1}\dots \xi _n^{-k_n} \ W_n(\vec {t};\xi _1,\dots ,\xi _n). \end{aligned}$$

#### Proof

Direct consequences of the definitions. $\square$

## Reformulation as spinor trans-series

### Motivation

Let us make some observations:

- A key ingredient in Hirota equation is that a shift $\vec {t}\mapsto \vec {t}+\vec {u} +[\xi ]$ is understood as a formal series in $u_k$s and in $\xi$, computed by the Taylor series formula.
- in Hirota equation, the Sato-shifted Tau functions always appear multiplied by an exponential, which is not a power series of $\xi$, instead it contains exponential of negative powers of $\xi$ (and which are useful to produce residues). Somehow, shifting is accompanied by an exponential term.
  Formal series multiplied by exponentials of negative powers are not formal series, they are called “**trans-series**” (see [30–32, 70, 75]).
- The insertion operator $\Delta$ is formally an operator of degree 0 in the filtration (indeed $\partial /\partial t_k$ has degree $-k$ while $\xi ^k$ has degree *k*), but is made of infinitely many terms. This means that associativity and commutativity of using $e^{\alpha \int \Delta }$ are not guaranteed. In some sense, such operators need to be defined outside of the ring ${\mathbb {C}}[[\Gamma ]]$ of graded formal series, they need to take into account exponential prefactors.
- Another observation is that Residues are defined for 1-forms, and they are invariant under change of variables only if one includes the Jacobian coming from the $d\xi$. In other words, the nature of the product of Tau functions inside the residue should be a 1-form, which means that each factor $\hat{{\mathcal {T}}}$ transforms as a power of $d\xi$, such that the product of the two factors transforms as $d\xi$. We shall redefine $\hat{{\mathcal {T}}}$ to include a power of $d\xi$; this will make $\hat{{\mathcal {T}}}$ a spinor (a differential with fractional power of $d\xi$), typically each $\hat{{\mathcal {T}}}$ will carry a half-integer power $\sqrt{d\xi }$.
- In actual realizations of integrable systems, for example as integrals (example matrix integrals in random matrix theory, see Section 7.1), Tau functions have asymptotic expansions that often are not convergent series, they are usually factorially divergent series, and they usually have an exponential prefactor, which is not a series in $\xi$. The good formalism to describe asymptotic expansions of exponential integrals or random matrix integrals is again the notion of trans-series rather than series. Moreover, the theory of resurgence [30–32, 70, 75] claims that whenever we have a divergent series, it should always be considered as trans-series.

All this suggest that the good framework for Tau functions should not be “series”, but rather “trans-series”, and moreover spinors (with a $\sqrt{d\xi }$).

Trans-series are linear combinations of series multiplied by some exponential. They have a rather different behavior than series, and in particular the Taylor formula and other operations cannot be applied naively. This shows that Hirota equations should better be reformulated in **spinor trans-series** rather than series.

### Tau function as trans-series

#### Definition 2.1

(*Tau function*) We define a Tau function ${\mathcal {T}}$ by the following functional properties: we say that ${\mathcal {T}}$ is a “Tau function” iff:

- 2.1 $$\begin{aligned} {\mathcal {T}}:= e^F \hat{{\mathcal {T}}}, \end{aligned}$$
  where $\hat{{\mathcal {T}}}(\vec {t}) \in {\mathbb {C}}[[\Gamma ]]$ is a graded formal series, and *F* is a function on $\Gamma$ homogeneous of degree 2:
  2.2 $$\begin{aligned} F(\lambda \vec {t}) = \lambda ^2 F(\vec {t}), \end{aligned}$$
  (but $$F\notin {\mathbb {C}}[[\Gamma ]]$$),
- for every divisor $D=\sum _{i=1}^\ell \alpha _i. z_i$ of degree $\deg D=0$:
  2.3 $$\begin{aligned} {\mathcal {T}}(\vec {t}+[D]) = {\mathcal {T}}(\vec {t}) e^{-\sum _{k\ge 1} t_k \sum _i \alpha _i \frac{z_i^{-k}}{k}} \prod _{i<j} E(z_i,z_j)^{\alpha _i\alpha _j} \ \frac{\hat{{\mathcal {T}}}(\vec {t}+[D])}{\hat{{\mathcal {T}}}(\vec {t})}, \end{aligned}$$
  where $E(z_i,z_j)$ is the Fay’s prime form [46]:
  2.4 $$\begin{aligned} E(z,z') := \frac{z-z'}{\sqrt{ dz dz'}} , \end{aligned}$$
  and where $\hat{{\mathcal {T}}}(\vec {t}+[D])$ is a graded formal series
  2.5 $$\begin{aligned} \hat{{\mathcal {T}}}(\vec {t}+[D]) \in {\mathbb {C}}[[z_1,\dots ,z_\ell ,\vec {t}]]. \end{aligned}$$
- It satisfies the Hirota equation (see Proposition 2.3), or its alternative Fay equations Theorem 2.2 or Theorem 3.3.

#### Proposition 2.1

The definition is consistent. With this definition, the Sato shift is associative (and commutative up to a phase): if *D* and $D'$ are two divisors of degree 0, we have

2.6 $$\begin{aligned} {\mathcal {T}}((\vec {t}+[D])+[D']) = {\mathcal {T}}(\vec {t}+([D]+[D'])). \end{aligned}$$

For $\sigma \in \mathfrak S_{\ell }$ a permutation, we denote $\sigma (D) = \sum _{i=1}^{\ell } \alpha _{\sigma (i)}.z_{\sigma (i)}$ the permuted divisor. We have

2.7 $$\begin{aligned} {\mathcal {T}}(\vec {t}+[\sigma (D)]) = e^{\pi i\sum _{i<j, \ \sigma (i)>\sigma (j)}\alpha _i\alpha _j } \ \ {\mathcal {T}}(\vec {t}+[D]) . \end{aligned}$$

If all charges $\alpha _i\in 2\mathbb Z+1$ are odd, the Tau function is fermionic, it obeys the Pauli symmetry, namely

2.8 $$\begin{aligned} {\mathcal {T}}(\vec {t}+[\sigma (D)]) = (-1)^\sigma \ {\mathcal {T}}(\vec {t}+[D]) . \end{aligned}$$

#### Proof

2.9 $$\begin{aligned} {\mathcal {T}}((\vec {t}+[D])+[D'])= &   {\mathcal {T}}(\vec {t}+[D]) e^{-\sum _k (t_k+\sum _j \alpha _j z_j^k) \sum _i \alpha '_i \frac{{z'_i}^{-k}}{k}} \\  &   \prod _{i<j} E(z'_i,z'_j)^{\alpha '_i\alpha '_j} \ \frac{\hat{{\mathcal {T}}}(\vec {t}+[D]+[D'])}{\hat{{\mathcal {T}}}(\vec {t}+[D])} \\= &   {\mathcal {T}}(\vec {t}+[D]) \prod _i e^{-\sum _k t_k \alpha _i' \frac{{z'_i}^{-k}}{k}} \prod _{i,j} e^{-\sum _k \alpha '_i\alpha _j z_j^k\frac{{z'_i}^{-k}}{k}} \\  &   \prod _{i<j} E(z'_i,z'_j)^{\alpha '_i\alpha '_j} \ \frac{\hat{{\mathcal {T}}}(\vec {t}+[D]+[D'])}{\hat{{\mathcal {T}}}(\vec {t}+[D])} \\= &   {\mathcal {T}}(\vec {t}+[D]) \prod _i e^{-\sum _k t_k \alpha _i' \frac{{z'_i}^{-k}}{k}} \prod _{i,j} e^{\alpha '_i\alpha _j \ln (1-z_j/z'_i)} \\  &   \prod _{i<j} E(z'_i,z'_j)^{\alpha '_i\alpha '_j} \ \frac{\hat{{\mathcal {T}}}(\vec {t}+[D]+[D'])}{\hat{{\mathcal {T}}}(\vec {t}+[D])} \\= &   {\mathcal {T}}(\vec {t}+[D]) \prod _i e^{-\sum _k t_k \alpha _i' \frac{{z'_i}^{-k}}{k}} \prod _{i,j} (1-z_j/z'_i)^{\alpha '_i\alpha _j } \\  &   \prod _{i<j} E(z'_i,z'_j)^{\alpha '_i\alpha '_j} \ \frac{\hat{{\mathcal {T}}}(\vec {t}+[D]+[D'])}{\hat{{\mathcal {T}}}(\vec {t}+[D])} \\= &   {\mathcal {T}}(\vec {t}+[D])\ \prod _i z_i'^{-\alpha '_i \deg D} e^{-\sum _k \alpha _i' t_k \frac{{z'_i}^{-k}}{k}} \prod _{i,j} (z'_i-z_j)^{\alpha '_i\alpha _j } \\  &   \prod _{i<j} E(z'_i,z'_j)^{\alpha '_i\alpha '_j} \ \frac{\hat{{\mathcal {T}}}(\vec {t}+[D]+[D'])}{\hat{{\mathcal {T}}}(\vec {t}+[D])} \\= &   {\mathcal {T}}(\vec {t}) \prod _j e^{-\alpha _j \sum _k \frac{t_k}{k} z_j^{-k} } \ \prod _{i<j} E(z_i,z_j)^{\alpha _i \alpha _j} \frac{\hat{{\mathcal {T}}}(\vec {t}+[D])}{\hat{{\mathcal {T}}}(\vec {t})} \\  &   \prod _i e^{-\sum _k t_k \alpha _i' \frac{{z'_i}^{-k}}{k}} \prod _{i,j} E(z'_i,z_j)^{\alpha '_i\alpha _j } \\  &   \prod _i dz_i'^{\frac{1}{2} \alpha '_i \deg D}\prod _j dz_j^{\frac{1}{2} \alpha _j \deg D'} \\  &   \prod _{i<j} E(z'_i,z'_j)^{\alpha '_i\alpha '_j} \ \frac{\hat{{\mathcal {T}}}(\vec {t}+[D]+[D'])}{\hat{{\mathcal {T}}}(\vec {t}+[D])} \\= &   {\mathcal {T}}(\vec {t}) \prod _j e^{-\alpha _j \sum _k \frac{t_k}{k} z_j^{-k} } \prod _i e^{-\sum _k t_k \alpha _i' \frac{{z'_i}^{-k}}{k}} \\  &   \prod _{i<j} E(z_i,z_j)^{\alpha _i \alpha _j} \prod _{i,j} E(z'_i,z_j)^{\alpha '_i\alpha _j } \prod _{i<j} E(z'_i,z'_j)^{\alpha '_i\alpha '_j} \\  &   \ \frac{\hat{{\mathcal {T}}}(\vec {t}+[D]+[D'])}{\hat{{\mathcal {T}}}(\vec {t})} \\= &   {\mathcal {T}}(\vec {t} + ([D]+[D'])) \end{aligned}$$

which concludes the associativity.

Now let us consider a permutation $\sigma$. We have:

2.10 $$\begin{aligned} {\mathcal {T}}(\vec {t}+[\sigma (D)])={\mathcal {T}}(\vec {t}) e^{-\sum _k t_k \sum _i \alpha _{\sigma (i)} \frac{z_{\sigma (i)}^{-k}}{k}} \prod _{i<j} E(z_{\sigma (i)},z_{\sigma (j)})^{\alpha _{\sigma (i)}\alpha _{\sigma (j)}} \ \frac{\hat{{\mathcal {T}}}(\vec {t}+[D])}{\hat{{\mathcal {T}}}(\vec {t})}, \end{aligned}$$

furthermore

2.11 $$\begin{aligned} \prod _{i<j} E(z_{\sigma (i)},z_{\sigma (j)})^{\alpha _{\sigma (i)}\alpha _{\sigma (j)}}= &   \prod _{i<j, \sigma (i)>\sigma (j)} E(z_{\sigma (i)},z_{\sigma (j)})^{\alpha _{\sigma (i)}\alpha _{\sigma (j)}} \nonumber \\  &   \prod _{i<j, \sigma (i)<\sigma (j)} E(z_{\sigma (i)},z_{\sigma (j)})^{\alpha _{\sigma (i)}\alpha _{\sigma (j)}} \nonumber \\= &   \prod _{i<j, \sigma (i)>\sigma (j)} (-E(z_{\sigma (j)},z_{\sigma (i)}))^{\alpha _{\sigma (j)}\alpha _{\sigma (i)}} \nonumber \\    &   \prod _{i<j, \sigma (i)<\sigma (j)} E(z_{\sigma (i)},z_{\sigma (j)})^{\alpha _{\sigma (i)}\alpha _{\sigma (j)}} \nonumber \\= &   e^{\pi i\sum _{i<j, \ \sigma (i)>\sigma (j)}\alpha _i\alpha _j }\nonumber \\    &   \prod _{i\ne j, \sigma (i)<\sigma (j)} E(z_{\sigma (i)},z_{\sigma (j)})^{\alpha _{\sigma (i)}\alpha _{\sigma (j)}} \nonumber \\= &   e^{\pi i\sum _{i<j, \ \sigma (i)>\sigma (j)}\alpha _i\alpha _j }\prod _{i<j} E(z_i,z_j)^{\alpha _i\alpha _j}. \end{aligned}$$

Therefore,

2.12 $$\begin{aligned} {\mathcal {T}}(\vec {t}+[\sigma (D)]) = e^{\pi i s_\sigma } \ \ {\mathcal {T}}(\vec {t}+[D]), \end{aligned}$$

where

2.13 $$\begin{aligned} s_\sigma = \sum _{i<j, \ \sigma (i)>\sigma (j)}\alpha _i\alpha _j . \end{aligned}$$

Notice that the parity of $s_\sigma$ is the same as the parity of only the terms with odd $\alpha _i$s.

Assume that all $\alpha _i$s are odd, i.e., $\alpha _i \equiv 1 \mod 2$. Then, we have

2.14 $$\begin{aligned} s_\sigma \equiv \sum _{i<j, \ \sigma (i)>\sigma (j)} 1 \mod 2, \end{aligned}$$

and we recognize the number of inversions of $\sigma$. It is well known that the parity of the number of inversions is the signature of $\sigma$; therefore, when all $\alpha _i$ are odd, we have

2.15 $$\begin{aligned} {\mathcal {T}}(\vec {t}+[\sigma (D)]) = (-1)^{s_\sigma } \ \ {\mathcal {T}}(\vec {t}+[D]). \end{aligned}$$

$$\square $$

#### Proposition 2.2

2.16 $$\begin{aligned} F(\vec {t}+D) = F(\vec {t}) -\sum _{k=1}^\infty \frac{1}{k} t_k {\operatorname {Tr}}D^{-k} + \sum _{i<j} \alpha _i\alpha _j \ln {E(z_i,z_j)} \end{aligned}$$

2.17 $$\begin{aligned} \Delta _\xi F = -dV_{\vec {t}}(\xi ). \end{aligned}$$

2.18 $$\begin{aligned} \Delta _\xi \ln {\mathcal {T}}= W_1(\xi ). \end{aligned}$$

2.19 $$\begin{aligned} \Delta _{\xi _1}\dots \Delta _{\xi _n} \ln {\mathcal {T}}= W_n(\xi _1,\dots ,\xi _n). \end{aligned}$$

#### Proof

Let’s take a zero degree divisor, we have:

2.20 $$\begin{aligned} \frac{{\mathcal {T}}(\vec {t}+D)}{{\mathcal {T}}(\vec {t})}=e ^{F(\vec {t}+ D)-F(\vec {t})} \,\frac{\hat{{\mathcal {T}}}(\vec {t}+D)}{\hat{{\mathcal {T}}}(\vec {t})}. \end{aligned}$$

From Eq. (2.3), we write

2.21 $$\begin{aligned} F(\vec {t}+D)-F(\vec {t})=-\sum _{k\ge 1}t_k \sum _i \alpha _i \frac{z_i^{-k}}{k}+\sum _{i<j} \alpha _i \alpha _j \ln E(z_i,z_j). \end{aligned}$$

For $$k>0$$, $${\operatorname {Tr}}D^{-k}=\sum _{i=1}^{l} \alpha _i z_i^{-k}$$, therefore

2.22 $$\begin{aligned} F(\vec {t}+D) = F(\vec {t}) -\sum _{k=1}^\infty \frac{1}{k} t_k {\operatorname {Tr}}D^{-k} + \sum _{i<j} \alpha _i\alpha _j \ln {E(z_i,z_j)}. \end{aligned}$$

Consider the following divisor

2.23 $$\begin{aligned} D=\alpha [\xi ]-\alpha [\xi _1], \end{aligned}$$

where $\xi _1$ is a fixed complex number. *D* is a zero degree divisor, and therefore, Eq. (2.16) gives

2.24 $$\begin{aligned} F(\vec {t}+ \alpha [\xi ]-\alpha [\xi _1])=F(\vec {t})- \sum _{k=1}^{\infty }\frac{1}{k}t_k\left( \alpha \xi ^{-k}-\alpha \xi _1^{-k}\right) -\alpha ^2\ln E(\xi ,\xi _1). \end{aligned}$$

Using Eq. (1.30) of Lemma 1.1, we get

2.25 $$\begin{aligned} \Delta _{\xi } F=\sum _{k=1}^{\infty } t_k \xi ^{-k-1} d\xi =-dV_{\vec {t}} (\xi ). \end{aligned}$$

Equation (2.3) implies

2.26 $$\begin{aligned} \begin{aligned} \Delta _\xi \ln {\mathcal {T}}&=\Delta _\xi \ln \hat{{\mathcal {T}}}+\Delta _\xi F\\&=\Delta _\xi \ln \hat{{\mathcal {T}}}-dV_{\vec {t}} (\xi )\\&=W_1(\vec {t}, \xi ). \end{aligned} \end{aligned}$$

Let $\xi _1$, $\xi _2$ $\in {\mathbb {C}}$, we have

2.27 $$\begin{aligned} \Delta _{\xi _2} \otimes \Delta _{\xi _1} \ln {\mathcal {T}}=\Delta _{\xi _2} \otimes \Delta _{\xi _1} \ln \hat{{\mathcal {T}}}+\Delta _{\xi _2} \otimes \Delta _{\xi _1} F. \end{aligned}$$

Using Eq. (1.37), we write

2.28 $$\begin{aligned} \begin{aligned} \Delta _{\xi _2} \otimes \Delta _{\xi _1} F&=\Delta _{\xi _2} (\Delta _{\xi _1} F)\\&=\Delta _{\xi _2} (-dV_{\vec {t}} (\xi ))\\&= \frac{d\xi _1 \otimes d\xi _2}{(\xi _1-\xi _2)^2}. \end{aligned} \end{aligned}$$

From this last equality, we can see that the quantity $\Delta _{\xi _2} \otimes \Delta _{\xi _1} F$ is independent of $\vec {t}$, and therefore, for $n>2$ we get

2.29 $$\begin{aligned} \Delta _{\xi _1}\dots \Delta _{\xi _n} F=0, \end{aligned}$$

thus Eq. (2.19) follows. $\square$

#### Remark 2.1

(*F*) In some sense, if one could define some negative times $t_{-k}$, whose Sato shift would be $t_{-k}\rightarrow t_{-k}+{\operatorname {Tr}}D^{-k}$, one would have that $F=\frac{1}{2} \sum _{k=1}^\infty \frac{1}{k} t_k t_{-k}$. However, this is ill-defined here; it can make sense only if one would introduce a pairing between $C[[t_1,t_2,\dots ]]$ and $C[[t_{-1},t_{-2},\dots ]]$, and this can be done case by case in various integrable systems. See the function $F_0$ in [36, 39], and that it requires a choice of polarization. So here instead, we define *F* by its functional property Eq. (2.16).

#### Proposition 2.3

(Hirota) The Hirota equation can be written

2.30 $$\begin{aligned} \mathop {{\textrm{Res}}}_{\xi \rightarrow 0} \,{\mathcal {T}}(\vec {t} + \vec {u}+ [\xi ]) {\mathcal {T}}(\vec {t}-\vec {u} - [\xi ]) = 0 \quad \text {in } {\mathbb {C}}[[u_1,u_2,\dots ]] \end{aligned}$$

or equivalently $$\forall \mu =(\mu _1,\dots ,\mu _\ell )$$

2.31 $$\begin{aligned} \mathop {{\textrm{Res}}}_{\xi \rightarrow 0}\, [u_1^{\mu _1} u_2^{\mu _2}\dots u_n^{\mu _n}] \left( {\mathcal {T}}(\vec {t} + \vec {u}+ [\xi ]) {\mathcal {T}}(\vec {t}-\vec {u} - [\xi ]) \right) = 0 \end{aligned}$$

or equivalently

2.32 $$\begin{aligned} \mathop {{\textrm{Res}}}_{\xi \rightarrow 0}\, \mathop {{\textrm{Res}}}_{u_1\rightarrow 0}\dots \mathop {{\textrm{Res}}}_{u_n\rightarrow 0}\, \frac{du_1}{u_1^{\mu _1+1}} \frac{du_2}{u_2^{\mu _2+1}}\dots \frac{du_n}{u_n^{\mu _n+1}} \left( {\mathcal {T}}(\vec {t} + \vec {u}+ [\xi ]) {\mathcal {T}}(\vec {t}-\vec {u} - [\xi ]) \right) = 0.\nonumber \\ \end{aligned}$$

#### Proof

Simple rewriting since our definition of ${\mathcal {T}}$ consisted in including the exponential and the $d\xi$ in the definition of ${\mathcal {T}}$. $\square$

### Hirota with divisors

The goal is to choose $\vec {u}$ as the Sato vector of a divisor *D* of degree $\deg D=-1$ (so that $\vec {u}+[\xi ]$ is a divisor of degree 0):

2.33 $$\begin{aligned} \vec {u} = [D] = \sum _{j=1}^\ell \alpha _j [z_j], \quad \deg D = \sum _{j=1}^\ell \alpha _j = -1, \end{aligned}$$

and replace in Hirota equations the formal series expansion in powers of $u^\mu$ as formal series in powers of $z_j$s.

#### Theorem 2.1

(Hirota with divisors) We have the equivalence betweenNow assume

- 2.34 $$\begin{aligned} 0=\mathop {{\textrm{Res}}}_{\xi \rightarrow 0}\,\frac{d\xi }{\xi ^2} \ \hat{{\mathcal {T}}}(\vec {t}+\vec {u}+[\xi ]) \hat{{\mathcal {T}}}(\vec {t}-\vec {u}-[\xi ]) e^{-2\sum _k \frac{u_k}{k} \xi ^{-k}} \quad \text {in } {\mathbb {C}}[[u_1,u_2,\dots ]] \end{aligned}$$
  or equivalently
  2.35 $$\begin{aligned} \forall \mu \ , \quad 0=\mathop {{\textrm{Res}}}_{\xi \rightarrow 0}\,\frac{d\xi }{\xi ^2} [\vec {u}^\mu ] \hat{{\mathcal {T}}}(\vec {t}+\vec {u}+[\xi ]) \hat{{\mathcal {T}}}(\vec {t}-\vec {u}-[\xi ]) e^{-2\sum _k \frac{u_k}{k} \xi ^{-k}} \end{aligned}$$
- and
  2.36 $$\begin{aligned}  &   \forall \ell \ge 1, \ \forall \alpha _1,\dots ,\alpha _{\ell } \text { such that } \sum _{i=1}^\ell \alpha _i=-1\nonumber \\  &   0 =\mathop {{\textrm{Res}}}_{\xi \rightarrow 0}\,\frac{d\xi }{\xi ^2} \hat{{\mathcal {T}}}(\vec {t}+[D]+[\xi ]) \hat{{\mathcal {T}}}(\vec {t}-[D]-[\xi ]) \prod _{j=1}^\ell (1-z_j/\xi )^{2\alpha _j} \nonumber \\  &   \quad \text {in } {\mathbb {C}}[[z_1,\dots ,z_\ell ]] \text { where } D = \sum _{j=1}^\ell \alpha _j.z_j. \end{aligned}$$

In other words, Hirota equation is equivalent to (2.36).

#### Proof

Start by assuming (2.35). Let $D = \sum _{j=1}^\ell \alpha _j.z_j$ a divisor of degree $-1$. We have

2.37 $$\begin{aligned} \hat{{\mathcal {T}}}(\vec {t}+[D]+[\xi ]) \hat{{\mathcal {T}}}(\vec {t}-[D]-[\xi ]) \prod _{j=1}^\ell (1-z_j/\xi )^{2\alpha _j} \in {\mathbb {C}}[\xi ,\xi ^{-1}][[z_1,\dots ,z_\ell ]], \end{aligned}$$

and thus

2.38 $$\begin{aligned} \mathop {{\textrm{Res}}}_{\xi \rightarrow 0}\,\frac{d\xi }{\xi ^2} \hat{{\mathcal {T}}}(\vec {t}+[D]+[\xi ]) \hat{{\mathcal {T}}}(\vec {t}-[D]-[\xi ]) \prod _{j=1}^\ell (1-z_j/\xi )^{2\alpha _j} \in {\mathbb {C}}[[z_1,\dots ,z_\ell ]]. \end{aligned}$$

In ${\mathbb {C}}[[z_1,\dots ,z_\ell ]]$ we have $(1-z_j/\xi )^{2\alpha _j} = \exp (2\alpha _j \ln {(1-z_j/\xi )})$, and thus this can be rewritten

2.39 $$\begin{aligned} \mathop {{\textrm{Res}}}_{\xi \rightarrow 0}\,\frac{d\xi }{\xi ^2} \hat{{\mathcal {T}}}(\vec {t}+[D]+[\xi ]) \hat{{\mathcal {T}}}(\vec {t}-[D]-[\xi ]) e^{-2\sum _{k\ge 1} \frac{\xi ^{-k}}{k} {\operatorname {Tr}}D^k} \in {\mathbb {C}}[[z_1,\dots ,z_\ell ]], \end{aligned}$$

and writing $u_k={\operatorname {Tr}}D^k$, it can be rewritten

2.40 $$\begin{aligned} \mathop {{\textrm{Res}}}_{\xi \rightarrow 0}\,\frac{d\xi }{\xi ^2} \hat{{\mathcal {T}}}(\vec {t}+\vec {u}+[\xi ]) \hat{{\mathcal {T}}}(\vec {t}-\vec {u}-[\xi ]) e^{-2\sum _k \frac{\xi ^{-k}}{k} u_k} \in {\mathbb {C}}[[u_1,u_2,\dots ]]. \end{aligned}$$

Thanks to (2.35), it is vanishing, and this implies (2.36).

Now assume (2.36). Let $\mu =(\mu _1,\dots ,\mu _\ell )$. Our first step is to construct, for every $\ell$-upple $u_1,\dots ,u_\ell$, a divisor *D* of degree $-1$, such that for every $k\le \ell$ we have ${\operatorname {Tr}}D^k=u_k$.

For that, let $N=2\ell +1$, let $\alpha _1=\dots =\alpha _\ell =1$ and $\alpha _{\ell +1}=\dots =\alpha _{2\ell +1}=-1$. Let $z_{\ell +j} = 1$ for $j=1,\dots ,\ell +1$.

Let

2.41 $$\begin{aligned} P(u_1,\dots ,u_\ell ;t)= &   \sum _{m} \frac{(-1)^m}{m!} \sum _{k_1,\dots ,k_m, \ k_i \ge 1, \ \sum _{i=1}^m k_i \le \ell } \prod _{i=1}^m \frac{(\ell +1) u_1^k +u_{k_i}}{k_i} \ t^{\ell -\sum _{i=1}^m k_i} \\= &   \left[ t^\ell \ e^{-\sum _{k=1}^\ell \frac{t^{-k}}{k} (u_k+(\ell +1)u_1^k)} \right] _+, \end{aligned}$$

where the second line means that we expand the exponential as a series of 1/*t* and keep only the non-negative powers of *t*. $P(u_1,\dots ,u_\ell ;t)$ is thus a monic polynomial of *t* of degree $\ell$. It has $\ell$ zeros denoted $z_1,\dots ,z_\ell$. They are such that (this is the Newton’s inversion formula)

2.42 $$\begin{aligned} \forall k=1,\dots ,\ell \qquad \sum _{i=1}^\ell z_i^k = (\ell +1)u_1^k + u_k. \end{aligned}$$

In other words, the divisor $D=\sum _{i=1}^\ell [z_i] - (\ell +1) [u_1]$ has degree $\deg D=-1$ and is such that

2.43 $$\begin{aligned} \forall k=1,\dots ,\ell \qquad {\operatorname {Tr}}D^k = u_k. \end{aligned}$$

This defines a map:

2.44 $$\begin{aligned} \phi :  &   {\mathbb {C}}^{\ell } \rightarrow \{\text {divisors of degree } -1\} \\  &   \quad (u_1,\dots ,u_\ell ) D = \sum _{i=1}^\ell [z_i] - (\ell +1) [u_1]. \end{aligned}$$

Moreover, $\phi$ respects the grading:

2.45 $$\begin{aligned} \phi (\lambda u_1 , \lambda ^2 u_2,\dots ,\lambda ^{\ell } u_l) = \lambda \phi (u_1,u_2,\dots ,u_\ell ). \end{aligned}$$

By definition, we have (where $\phi (u_1,\dots ,u_\ell )=D=\sum _{i=1}^\ell [z_i] - (\ell +1)[u_1]$):

2.46 $$\begin{aligned}  &   \mathop {{\textrm{Res}}}_{\xi \rightarrow 0}\,\frac{d\xi }{\xi ^2} [\vec {u}^\mu ] \hat{{\mathcal {T}}}(\vec {t}+\vec {u}+[\xi ]) \hat{{\mathcal {T}}}(\vec {t}-\vec {u}-[\xi ]) e^{-2\sum _k \frac{u_k}{k} \xi ^{-k}} \nonumber \\    &   \mathop {{\textrm{Res}}}_{\xi \rightarrow 0}\,\frac{d\xi }{\xi ^2} [\vec {u}^\mu ] \hat{{\mathcal {T}}}(\vec {t}+[\phi (\vec {u})]+[\xi ]) \hat{{\mathcal {T}}}(\vec {t}-[\phi (\vec {u})]-[\xi ]) \frac{\prod _{i=1}^{\ell } (1-z_j/\xi )^2}{(1-u_1/\xi )^{2(\ell +1)}}.\nonumber \\ \end{aligned}$$

From (2.36), we know that

2.47 $$\begin{aligned}  &   \mathop {{\textrm{Res}}}_{\xi \rightarrow 0}\,\frac{d\xi }{\xi ^2} \hat{{\mathcal {T}}}(\vec {t}+[\phi (\vec {u})]+[\xi ]) \hat{{\mathcal {T}}}(\vec {t}-[\phi (\vec {u})]-[\xi ]) \frac{\prod _{i=1}^{\ell } (1-z_j/\xi )^2}{(1-u_1/\xi )^{2(\ell +1)}}\nonumber \\  &   \quad = 0 \quad \text {in } {\mathbb {C}}[[u_1,z_1,\dots ,z_\ell ]]. \end{aligned}$$

This implies that it vanishes in ${\mathbb {C}}[[u_1,u_2,\dots ,u_\ell ]]$, and this proves (2.35). $\square$

With ${\mathcal {T}}$ this is reformulated as:

#### Theorem 2.2

(Hirota with divisors) ${\mathcal {T}}$ satisfies Hirota equation is equivalent to:

$$\forall \ell \ge 1, \ \forall \alpha _1,\dots ,\alpha _{\ell } \text { such that } \sum _{i=1}^\ell \alpha _i=-1$$

2.48 $$\begin{aligned} 0 =\mathop {{\textrm{Res}}}_{\xi \rightarrow 0}\, {\mathcal {T}}(\vec {t}+[D]+[\xi ]) {\mathcal {T}}(\vec {t}-[D]-[\xi ]) \quad \text {in } {\mathbb {C}}[[z_1,\dots ,z_\ell ]] \text { where } D = \sum _{j=1}^\ell \alpha _j [z_j]. \end{aligned}$$

The following lemma to compute residues of formal series will be very useful

#### Lemma 2.1

(Residues of formal series) If $f(z) = \sum _{j=0}^\infty \frac{f_j}{j!} z^j \in {\mathbb {C}}[[z]]$ is a formal series, we have

2.49 $$\begin{aligned} \mathop {{\textrm{Res}}}_0 \,\frac{d\xi }{\xi } f(\xi ) \ (1-z/\xi )^{-1} = f(z) \quad \text {in } {\mathbb {C}}[[z]], \end{aligned}$$

and more generally

2.50 $$\begin{aligned} \mathop {{\textrm{Res}}}_0 \,\frac{d\xi }{\xi ^{n+1}} f(\xi ) \ (1-z/\xi )^{-n-1} = \frac{1}{n!} f^{(n)}(z) \quad \text {in } {\mathbb {C}}[[z]], \end{aligned}$$

and this vanishes if $n<0$.

In other words, the formulas for residues of formal series are the same as if *f*(*z*) were an analytic function in a neighborhood of $z=0$.

#### Proof

2.51 $$\begin{aligned} \mathop {{\textrm{Res}}}_0 \,\frac{d\xi }{\xi ^{n+1}} f(\xi ) \ (1-z/\xi )^{-n-1}= &   \mathop {{\textrm{Res}}}_0\, \frac{d\xi }{\xi ^{n+1}} \left( \sum _{j=0}^\infty \frac{f_j}{j!} \xi ^j \right) \left( \sum _{k=0}^\infty \frac{(n+k)!}{k! n!} \frac{z^k}{\xi ^k} \right) \\= &   \sum _{k=0}^\infty \frac{f_{n+k}}{(n+k)!} \frac{(n+k)!}{k! n!} z^k \\= &   \frac{1}{n!} \sum _{k=0}^\infty \frac{f_{n+k}}{k!} z^k \\= &   \frac{1}{n!} f^{(n)}(z) \quad \text {in } {\mathbb {C}}[[z]]. \end{aligned}$$

$$\square $$

## Fay identities

Let us apply the divisor formulation of Hirota in the case of a divisor with four points of the form

3.1 $$\begin{aligned} {[}D] = \frac{1}{2}([z_1]-[z_2]-[\tilde{z}_1]-[\tilde{z}_2]) \end{aligned}$$

(which is indeed of degree $\deg D=-1$).

### Theorem 3.1

(Fay identities $n=2$) Hirota equation implies:

3.2 $$\begin{aligned}  &   \frac{(z_1-z_2)(\tilde{z}_1-\tilde{z}_2)}{(\tilde{z}_1-z_2)(\tilde{z}_2-z_2)(z_1-\tilde{z}_1)(z_1-\tilde{z}_2)} \ \frac{\hat{{\mathcal {T}}}(\vec {\tilde{t}} +[z_1]-[\tilde{z}_1]+[z_2]-[\tilde{z}_2])}{\hat{{\mathcal {T}}}(\vec {\tilde{t}})} \nonumber \\  &   \quad = \ \frac{\hat{{\mathcal {T}}}(\vec {\tilde{t}} +[z_1]-[\tilde{z}_2])}{(z_1-\tilde{z}_2)\hat{{\mathcal {T}}}(\vec {\tilde{t}})} \ \frac{\hat{{\mathcal {T}}}(\vec {\tilde{t}} +[z_2]-[\tilde{z}_1])}{(z_2-\tilde{z}_1)\hat{{\mathcal {T}}}(\vec {\tilde{t}})} - \ \frac{\hat{{\mathcal {T}}}(\vec {\tilde{t}} +[z_1]-[\tilde{z}_1])}{(z_1-\tilde{z}_1)\hat{{\mathcal {T}}}(\vec {\tilde{t}})} \ \frac{\hat{{\mathcal {T}}}(\vec {\tilde{t}} +[z_2]-[\tilde{z}_2])}{(z_2-\tilde{z}_2)\hat{{\mathcal {T}}}(\vec {\tilde{t}})}\nonumber \\ \end{aligned}$$

or equivalently, with ${\mathcal {T}}$ instead of $\hat{{\mathcal {T}}}$:

3.3 $$\begin{aligned}  &   \frac{{\mathcal {T}}(\vec {\tilde{t}} +[z_1]-[\tilde{z}_1]+[z_2]-[\tilde{z}_2])}{{\mathcal {T}}(\vec {\tilde{t}})} \nonumber \\  &   \quad = \frac{{\mathcal {T}}(\vec {\tilde{t}} +[z_1]-[\tilde{z}_1])}{{\mathcal {T}}(\vec {\tilde{t}})} \ \frac{{\mathcal {T}}(\vec {\tilde{t}} +[z_2]-[\tilde{z}_2])}{{\mathcal {T}}(\vec {\tilde{t}})} \ - \frac{{\mathcal {T}}(\vec {\tilde{t}} +[z_1]-[\tilde{z}_2])}{{\mathcal {T}}(\vec {\tilde{t}})} \ \frac{{\mathcal {T}}(\vec {\tilde{t}} +[z_2]-[\tilde{z}_1])}{{\mathcal {T}}(\vec {\tilde{t}})} \nonumber \\ \end{aligned}$$

or equivalently, as a determinantal formula

3.4 $$\begin{aligned} \frac{{\mathcal {T}}(\vec {\tilde{t}} +[z_1]-[\tilde{z}_1]+[z_2]-[\tilde{z}_2])}{{\mathcal {T}}(\vec {\tilde{t}})} = \det _{1\le i,j\le 2} \left( \frac{{\mathcal {T}}(\vec {\tilde{t}} +[z_i]-[\tilde{z}_j])}{{\mathcal {T}}(\vec {\tilde{t}})} \right) \ . \end{aligned}$$

All these equalities hold as formal series in ${\mathbb {C}}[[z_1,z_2,\tilde{z}_1,\tilde{z}_2]]$.

### Proof

Choose

3.5 $$\begin{aligned} [D] = \frac{1}{2}([z_1]-[z_2]-[\tilde{z}_1]-[\tilde{z}_2]), \end{aligned}$$

and

3.6 $$\begin{aligned} \vec {\tilde{t}} = \vec {t} -[D]-[z_2]. \end{aligned}$$

Hirota equation and Lemma 2.1 imply

3.7 $$\begin{aligned} 0= &   {\textrm{Res}} \frac{(z_1-\xi )d\xi }{(z_2-\xi )(\tilde{z}_1-\xi )(\tilde{z}_2-\xi )} \ \hat{{\mathcal {T}}}(\vec {\tilde{t}} +[z_1]-[\tilde{z}_1]-[\tilde{z}_2]+[\xi ]) \hat{{\mathcal {T}}}(\vec {\tilde{t}} +[z_2]-[\xi ]) \nonumber \\  = &   \frac{(z_1-z_2)}{(\tilde{z}_1-z_2)(\tilde{z}_2-z_2)} \ \hat{{\mathcal {T}}}(\vec {\tilde{t}} +[z_1]-[\tilde{z}_1]+[z_2]-[\tilde{z}_2]) \hat{{\mathcal {T}}}(\vec {\tilde{t}}) \nonumber \\    &   + \frac{(z_1-\tilde{z}_1)}{(z_2-\tilde{z}_1)(\tilde{z}_2-\tilde{z}_1)} \ \hat{{\mathcal {T}}}(\vec {\tilde{t}} +[z_1]-[\tilde{z}_2]) \hat{{\mathcal {T}}}(\vec {\tilde{t}} +[z_2]-[\tilde{z}_1]) \nonumber \\    &   + \frac{(z_1-\tilde{z}_2)}{(z_2-\tilde{z}_2)(\tilde{z}_1-\tilde{z}_2)} \ \hat{{\mathcal {T}}}(\vec {\tilde{t}} +[z_1]-[\tilde{z}_1]) \hat{{\mathcal {T}}}(\vec {\tilde{t}} +[z_2]-[\tilde{z}_2]) \end{aligned}$$

$$\square $$

### Definition 3.1

(*Kernel*) We define:

3.8 $$\begin{aligned} K(\xi ,\xi ') := {\mathcal {T}}(\vec {t}+[\xi ']-[\xi ])/{{\mathcal {T}}(\vec {t})}. \end{aligned}$$

It is a bi-spinor; it behaves near coinciding points as

3.9 $$\begin{aligned} K(\xi ,\xi ') = \frac{\sqrt{d\xi } \otimes \sqrt{d\xi '}}{\xi '-\xi } \ (1+O(\xi '-\xi )). \end{aligned}$$

### Theorem 3.2

(Fay identities for all $n\ge 1$) Let $D=\sum _{i=1}^n [z_i]-[\tilde{z}_i]$ a supersymmetric divisor (a unitary divisor of degree 0). We have the determinantal formula:

3.10 $$\begin{aligned} \frac{{\mathcal {T}}(\vec {\tilde{t}} +[D])}{{\mathcal {T}}(\vec {\tilde{t}})} = \det _{1\le i,j\le n} \left( \frac{{\mathcal {T}}(\vec {\tilde{t}} +[z_j]-[\tilde{z}_i])}{{\mathcal {T}}(\vec {\tilde{t}})} \right) \ = \det K(\tilde{\xi }_i,\xi _j) \end{aligned}$$

as formal series in ${\mathbb {C}}[[z_1,\dots ,z_n,\tilde{z}_1,\dots ,\tilde{z}_n ]]$.

### Proof

We shall proceed by recursion on *n*. We know that this holds for $n=1$ and $n=2$. Let $n\ge 3$. Let us write:

3.11 $$\begin{aligned} D'_i=\sum _{j=2}^n [z_j]-\sum _{j\ne i} [\tilde{z}_j] = D-[z_1]+[\tilde{z}_i]. \end{aligned}$$

Hirota equation gives:

3.12 $$\begin{aligned} 0= &   \mathop {{\textrm{Res}}}_{0}\, \frac{d\xi }{z_1-\xi } \frac{\prod _{i=2}^n (\xi -z_i)}{\prod _{i=1}^n (\xi -\tilde{z}_i)} \ \hat{{\mathcal {T}}}(\vec {t} + [z_1]-[\xi ]) \hat{{\mathcal {T}}}(\vec {t} - [\tilde{z}_1]+[\xi ] + [D'_1]) \\= &   - \ \frac{\prod _{i=2}^n (z_1-z_i)}{\prod _{i=1}^n (z_1-\tilde{z}_i)} \ \hat{{\mathcal {T}}}(\vec {t} ) \hat{{\mathcal {T}}}(\vec {t} +[D]) \\  &   + \sum _{i=1}^n \frac{1}{z_1-\tilde{z}_i} \frac{\prod _{j=2}^n (\tilde{z}_i-z_j)}{\prod _{j\ne i}^n (\tilde{z}_i-\tilde{z}_j)} \ \ \hat{{\mathcal {T}}}(\vec {t} +[z_1]-[\tilde{z}_i]) \hat{{\mathcal {T}}}(\vec {t} +[D'_i]). \end{aligned}$$

This gives

3.13 $$\begin{aligned}  &   \hat{{\mathcal {T}}}(\vec {t} ) \hat{{\mathcal {T}}}(\vec {t} +[D]) \nonumber \\  &   \quad = \frac{\prod _{j=1}^n (z_1-\tilde{z}_j)}{\prod _{j=2}^n (z_1-z_j)} \sum _{i=1}^n \frac{1}{z_1-\tilde{z}_i} \frac{\prod _{j=2}^n (\tilde{z}_i-z_j)}{\prod _{j\ne i}^n (\tilde{z}_i-\tilde{z}_j)} \ \ \hat{{\mathcal {T}}}(\vec {t} +[z_1]-[\tilde{z}_i]) \hat{{\mathcal {T}}}(\vec {t} +[D'_i]).\nonumber \\ \end{aligned}$$

or equivalently in terms of ${\mathcal {T}}$:

3.14 $$\begin{aligned} \frac{{\mathcal {T}}(\vec {t} +[D])}{{\mathcal {T}}(\vec {t} ) } = \sum _{i=1}^n (-1)^{i-1} \ \frac{{\mathcal {T}}(\vec {t} +[z_1]-[\tilde{z}_i])}{{\mathcal {T}}(\vec {t} ) } \ \frac{ {\mathcal {T}}(\vec {t} +[D'_i])}{{\mathcal {T}}(\vec {t} ) } . \end{aligned}$$

We compute the last factor with the recursion hypothesis:

3.15 $$\begin{aligned} \frac{{\mathcal {T}}(\vec {t} +[D])}{{\mathcal {T}}(\vec {t} ) }= &   \sum _{i=1}^n \frac{{\mathcal {T}}(\vec {t} +[z_1]-[\tilde{z}_i]) }{{\mathcal {T}}(\vec {t} ) } \sum _{\sigma \in \mathfrak S_{n} \, \ \sigma (1)=i} (-1)^\sigma \ \prod _{j=2}^n \frac{{\mathcal {T}}(\vec {t} +[z_j]-[\tilde{z}_{\sigma (j)}])}{{\mathcal {T}}(\vec {t} ) } \\= &   \sum _{\sigma \in \mathfrak S_{n} } (-1)^\sigma \ \prod _{j=1}^n \frac{{\mathcal {T}}(\vec {t} +[z_j]-[\tilde{z}_{\sigma (j)}])}{{\mathcal {T}}(\vec {t} ) }. \end{aligned}$$

$$\square $$

### Theorem 3.3

(Fay $\implies$ Hirota) If ${\mathcal {T}}$ is a functional as in Definition 2.1, that satisfies Fay determinantal formula for all *n* and all divisors, then it satisfies Hirota.

### Proof

(see [47]) Assume that Fay identities hold for all $n\ge 2$, i.e., all unitary divisor of degree 0.

For $$n=2$$, choosing

3.16 $$\begin{aligned} {[}D] = \frac{1}{2}([z_1]-[z_2]-[\tilde{z}_1]-[\tilde{z}_2]), \end{aligned}$$

and

3.17 $$\begin{aligned} \vec {\tilde{t}} = \vec {t} -[D]-[z_2]. \end{aligned}$$

We find

3.18 $$\begin{aligned}  &   \mathop {{\textrm{Res}}}_{0} \,\frac{(z_1-\xi )d\xi }{(z_2-\xi )(\tilde{z}_1-\xi )(\tilde{z}_2-\xi )} \ \hat{{\mathcal {T}}}(\vec {\tilde{t}} +[z_1]-[\tilde{z}_1]-[\tilde{z}_2]+[\xi ]) \hat{{\mathcal {T}}}(\vec {\tilde{t}} +[z_2]-[\xi ]) \\  &   \quad = \frac{(z_1-z_2)}{(\tilde{z}_1-z_2)(\tilde{z}_2-z_2)} \ \hat{{\mathcal {T}}}(\vec {\tilde{t}} +[z_1]-[\tilde{z}_1]+[z_2]-[\tilde{z}_2]) \hat{{\mathcal {T}}}(\vec {\tilde{t}}) \\  &   \qquad + \frac{(z_1-\tilde{z}_1)}{(z_2-\tilde{z}_1)(\tilde{z}_2-\tilde{z}_1)} \ \hat{{\mathcal {T}}}(\vec {\tilde{t}} +[z_1]-[\tilde{z}_2]) \hat{{\mathcal {T}}}(\vec {\tilde{t}} +[z_2]-[\tilde{z}_1]) \\  &   \qquad + \frac{(z_1-\tilde{z}_2)}{(z_2-\tilde{z}_2)(\tilde{z}_1-\tilde{z}_2)} \ \hat{{\mathcal {T}}}(\vec {\tilde{t}} +[z_1]-[\tilde{z}_1]) \hat{{\mathcal {T}}}(\vec {\tilde{t}} +[z_2]-[\tilde{z}_2]) \\  &   \quad = 0. \end{aligned}$$

Then, by a recursion on *n* (the same as in the proof of Theorem 3.2), we find

3.19 $$\begin{aligned}  &   \mathop {{\textrm{Res}}}_{0}\, \frac{d\xi }{z_1-\xi } \frac{\prod _{i=2}^n (\xi -z_i)}{\prod _{i=1}^n (\xi -\tilde{z}_i)} \ \frac{\hat{{\mathcal {T}}}(\vec {t} + [z_1]-[\xi ])}{{\mathcal {T}}(\vec {t})} \frac{\hat{{\mathcal {T}}}(\vec {t} - [\tilde{z}_1]+[\xi ] + [D'_1])}{{\mathcal {T}}(\vec {t})} \\  &   \quad \propto \left( \frac{{\mathcal {T}}(\vec {t} +[D])}{{\mathcal {T}}(\vec {t})} - \sum _\sigma (-1)^\sigma \prod _{i=1}^n K(\tilde{z}_i, z_{\sigma (i)})\right) \\  &   \quad = 0. \end{aligned}$$

Since this holds for all *n* and all *D*, this proves Hirota. $\square$

### Theorem 3.4

(Reproducing kernel) $K(\xi ,\xi ')$ is a self-reproducing kernel for the insertion operator:

3.20 $$\begin{aligned} \Delta _\xi \left( \frac{ {\mathcal {T}}(\vec {t}+[\xi ']-[\xi ''])}{{\mathcal {T}}(\vec {t})}\right) = - \ \frac{ {\mathcal {T}}(\vec {t}+[\xi ']-[\xi ])}{{\mathcal {T}}(\vec {t})} \ \frac{ {\mathcal {T}}(\vec {t}+[\xi ]-[\xi ''])}{{\mathcal {T}}(\vec {t})} \end{aligned}$$

or equivalently

3.21 $$\begin{aligned} \Delta _\xi K(\xi ',\xi '') = - K(\xi ',\xi )K(\xi ,\xi ''). \end{aligned}$$

### Proof

Take $D=\frac{1}{2}([\xi _1]-[\xi _2])-[\xi _0]$ in (2.36) and $\vec {\tilde{t}}=\vec {t} + \frac{1}{2}([\xi _1]-[\xi _2])$

3.22 $$\begin{aligned} 0= &   \mathop {{\textrm{Res}}}_0 \,\frac{(\xi -\xi _1)d\xi }{(\xi -\xi _2)(\xi -\xi _0)^2} \hat{{\mathcal {T}}}(\vec {\tilde{t}}+[\xi ]+\frac{1}{2}([\xi _1]-[\xi _2])-[\xi _0])\nonumber \\  &   \hat{{\mathcal {T}}}(\vec {\tilde{t}}-[\xi ]-\frac{1}{2}([\xi _1]-[\xi _2])+[\xi _0]) \nonumber \\  = &   \mathop {{\textrm{Res}}}_0\, \frac{(\xi -\xi _1)d\xi }{(\xi -\xi _2)(\xi -\xi _0)^2} \hat{{\mathcal {T}}}(\vec {t}+[\xi ]+[\xi _1]-[\xi _2]-[\xi _0])\hat{{\mathcal {T}}}(\vec {t}-[\xi ]+[\xi _0]) \nonumber \\  = &   \frac{(\xi _2-\xi _1)}{(\xi _2-\xi _0)^2} \hat{{\mathcal {T}}}(\vec {t}+[\xi _1]-[\xi _0])\hat{{\mathcal {T}}}(\vec {t}-[\xi _2]+[\xi _0]) \nonumber \\    &   + \frac{d}{d\xi } \left( \frac{(\xi -\xi _1)}{(\xi -\xi _2)} \hat{{\mathcal {T}}}(\vec {t}+[\xi ]+[\xi _1]-[\xi _2]-[\xi _0])\hat{{\mathcal {T}}}(\vec {t}-[\xi ]+[\xi _0]) \right) _{\xi =\xi _0} \nonumber \\  = &   \frac{(\xi _2-\xi _1)}{(\xi _2-\xi _0)^2} \hat{{\mathcal {T}}}(\vec {t}+[\xi _1]-[\xi _0])\hat{{\mathcal {T}}}(\vec {t}-[\xi _2]+[\xi _0]) \nonumber \\  &   + \frac{1}{(\xi _0-\xi _2)} \hat{{\mathcal {T}}}(\vec {t}+[\xi _1]-[\xi _2])\hat{{\mathcal {T}}}(\vec {t}) - \frac{(\xi _0-\xi _1)}{(\xi _0-\xi _2)^2} \hat{{\mathcal {T}}}(\vec {t}+[\xi _1]-[\xi _2])\hat{{\mathcal {T}}}(\vec {t}) \nonumber \\    &   + \frac{(\xi _0-\xi _1)}{(\xi _0-\xi _2)} \frac{d}{d\xi } \left( \hat{{\mathcal {T}}}(\vec {t}+[\xi ]+[\xi _1]-[\xi _2]-[\xi _0])\right) _{\xi =\xi _0}\hat{{\mathcal {T}}}(\vec {t}-[\xi ]+[\xi _0]) \nonumber \\    &   + \frac{(\xi _0-\xi _1)}{(\xi _0-\xi _2)} \hat{{\mathcal {T}}}(\vec {t}+[\xi ]+[\xi _1]-[\xi _2]-[\xi _0])\frac{d}{d\xi } \left( \hat{{\mathcal {T}}}(\vec {t}-[\xi ]+[\xi _0]) \right) _{\xi =\xi _0} \nonumber \\  = &   \frac{(\xi _2-\xi _1)}{(\xi _2-\xi _0)^2} \hat{{\mathcal {T}}}(\vec {t}+[\xi _1]-[\xi _0])\hat{{\mathcal {T}}}(\vec {t}-[\xi _2]+[\xi _0]) \nonumber \\    &   + \frac{\xi _1-\xi _2}{(\xi _0-\xi _2)^2} \hat{{\mathcal {T}}}(\vec {t}+[\xi _1]-[\xi _2])\hat{{\mathcal {T}}}(\vec {t}) \nonumber \\    &   + \frac{(\xi _0-\xi _1)}{(\xi _0-\xi _2)} \Delta _{\xi _0} \hat{{\mathcal {T}}}(\vec {t}+[\xi _1]-[\xi _2]) \ \hat{{\mathcal {T}}}(\vec {t}) \nonumber \\    &   - \frac{(\xi _0-\xi _1)}{(\xi _0-\xi _2)} \hat{{\mathcal {T}}}(\vec {t}+[\xi _1]-[\xi _2])\ \Delta _{\xi _0} \hat{{\mathcal {T}}}(\vec {t}). \end{aligned}$$

This implies

3.23 $$\begin{aligned}  &   \frac{(\xi _2-\xi _1)}{(\xi _0-\xi _1)(\xi _2-\xi _0)}\frac{\hat{{\mathcal {T}}}(\vec {t}+[\xi _1]-[\xi _0])}{\hat{{\mathcal {T}}}(\vec {t})} \ \frac{\hat{{\mathcal {T}}}(\vec {t}-[\xi _2]+[\xi _0])}{\hat{{\mathcal {T}}}(\vec {t})} \nonumber \\  &   \quad = \frac{\xi _1-\xi _2}{(\xi _0-\xi _2)(\xi _0-\xi _1)} \frac{\hat{{\mathcal {T}}}(\vec {t}+[\xi _1]-[\xi _2])}{\hat{{\mathcal {T}}}(\vec {t})} \nonumber \\  &   \qquad + \Delta _{\xi _0} \left( \frac{\hat{{\mathcal {T}}}(\vec {t}+[\xi _1]-[\xi _2])}{\hat{{\mathcal {T}}}(\vec {t})}\right) , \end{aligned}$$

i.e.,

3.24 $$\begin{aligned} \frac{{\mathcal {T}}(\vec {t}+[\xi _1]-[\xi _0])}{{\mathcal {T}}(\vec {t})} \ \frac{{\mathcal {T}}(\vec {t}-[\xi _2]+[\xi _0])}{{\mathcal {T}}(\vec {t})}= &   - \Delta _{\xi _0} \left( \frac{{\mathcal {T}}(\vec {t}+[\xi _1]-[\xi _2])}{{\mathcal {T}}(\vec {t})}\right) ,\nonumber \\ \end{aligned}$$

i.e.,

3.25 $$\begin{aligned} \Delta _{\xi _0} K(\xi _2,\xi _1) = - K(\xi _2,\xi _0) K(\xi _0,\xi _1). \end{aligned}$$

$$\square $$

A consequence is that the correlators $W_n$ are of the determinantal form as in [10]:

### Theorem 3.5

(determinantal formulas for $W_n$)

3.26 $$\begin{aligned} W_1(\xi ) = \lim _{\xi '\rightarrow \xi } K(\xi ,\xi ') - \frac{1}{E(\xi ',\xi )}. \end{aligned}$$

For $$n\ge 2$$

3.27 $$\begin{aligned} W_n(\xi _1,\dots ,\xi _n)= &   \sum _{\sigma \in \mathfrak S_n^{1-\text {cycle}}} (-1)^\sigma \prod _{i=1}^N \frac{{\mathcal {T}}(\vec {t} + [\xi _i]-[\xi _{\sigma (i)}])}{{\mathcal {T}}(\vec {t})} \nonumber \\= &   \sum _{\sigma \in \mathfrak S_n^{1-\text {cycle}}} (-1)^\sigma \prod _{i=1}^N K(\xi _{\sigma (i)},\xi _i). \end{aligned}$$

### Proof

For $n=1$, we have from (1.27) of Lemma 1.2:

3.28 $$\begin{aligned} \lim _{\xi '\rightarrow \xi } K(\xi ,\xi ') - \frac{1}{E(\xi ',\xi )}= &   \lim _{\xi '\rightarrow \xi } \frac{\hat{{\mathcal {T}}}(\vec {t}+[\xi ']-[\xi ])}{E(\xi ',\xi )\hat{{\mathcal {T}}}(\vec {t})} - \frac{1}{E(\xi ',\xi )} \\= &   \lim _{\xi '\rightarrow \xi } \frac{\hat{{\mathcal {T}}}(\vec {t}+[\xi ']-[\xi ])-\hat{{\mathcal {T}}}(\vec {t})}{E(\xi ',\xi )\hat{{\mathcal {T}}}(\vec {t})} \\= &   \frac{d\xi }{\hat{{\mathcal {T}}}(\vec {t})} \frac{d}{d\xi '}\left( \hat{{\mathcal {T}}}(\vec {t}+[\xi ']-[\xi ])\right) _{\xi '=\xi } \\= &   \frac{1}{\hat{{\mathcal {T}}}(\vec {t})} \Delta _\xi \hat{{\mathcal {T}}}(\vec {t}) \\= &   \Delta _\xi \ln \hat{{\mathcal {T}}}(\vec {t}) \\= &   W_1(\xi ). \end{aligned}$$

Then act with $\Delta _{\xi _2}$:

3.29 $$\begin{aligned} W_2(\xi _1,\xi _2)= &   \Delta _{\xi _2} W_1(\xi _1) \\= &   \Delta _{\xi _2} \left( \lim _{\xi '\rightarrow \xi _1} K(\xi _1,\xi ') - \frac{1}{E(\xi ',\xi _1)}\right) \\= &   \lim _{\xi '\rightarrow \xi _1} -K(\xi _1,\xi _2)K(\xi _2,\xi ') \\= &   -K(\xi _1,\xi _2)K(\xi _2,\xi _1). \end{aligned}$$

Then act recursively with $\Delta _{\xi _n}$: for $n+1\ge 3$, assume it is proved for *n*:

3.30 $$\begin{aligned} W_{n+1}(\xi _1,\dots ,\xi _n,\xi _{n+1})= &   \Delta _{\xi _{n+1}} W_{n}(\xi _1,\dots ,\xi _n) \nonumber \\  = &   \Delta _{\xi _{n+1}} \sum _{\sigma \in \mathfrak S_n} (-1)^{n-1} \prod _{i=1}^n K(x_i,x_{\sigma (i)}) \nonumber \\  = &   \sum _{\sigma \in \mathfrak S_n^{1-\text {cycle}}} (-1)^{n-1} \sum _{j=1}^n \Delta _{\xi _{n+1}}K(x_j,x_{\sigma (j)}) \prod _{i=1, \ i\ne j}^n K(x_i,x_{\sigma (i)}) \nonumber \\  = &   \sum _{\sigma \in \mathfrak S_n^{1-\text {cycle}}} (-1)^{n} \sum _{j=1}^n K(x_j,x_{n+1})K(x_{n+1},x_{\sigma (j)}) \nonumber \\    &   \prod _{i=1, \ i\ne j}^n K(x_i,x_{\sigma (i)}) \nonumber \\  = &   \sum _{j=1}^n \sum _{\sigma \in \mathfrak S_{n+1}^{1-\text {cycle}}, \ \sigma (j)=n+1} (-1)^{n} \prod _{i=1}^{n+1} K(x_i,x_{\sigma (i)}) \nonumber \\  = &   \sum _{\sigma \in \mathfrak S_{n+1}^{1-\text {cycle}}} (-1)^{n} \prod _{i=1}^{n+1} K(x_i,x_{\sigma (i)}) \end{aligned}$$

$$\square $$

### Miwa–Jimbo

Another consequence is that Tau function is automatically solutions of Miwa–Jimbo equations:

#### Theorem 3.6

(Miwa Jimbo)

3.31 $$\begin{aligned} \frac{\partial }{\partial t_k} \ln {\mathcal {T}}(\vec {t}) = \mathop {{\textrm{Res}}}_{\xi \rightarrow 0}\, \frac{1}{k} \xi ^{-k} W_1(\xi ). \end{aligned}$$

3.32 $$\begin{aligned} \frac{\partial ^n}{\partial t_{k_1}\dots \partial t_{k_n}} \ln {\mathcal {T}}(\vec {t}) = \mathop {{\textrm{Res}}}_{\xi _1\rightarrow 0}\,\dots \mathop {{\textrm{Res}}}_{\xi _n\rightarrow 0}\, \frac{\xi _1^{-k_1}\dots \xi _n^{-k_n}}{k_1\dots k_n} W_n(\xi _1,\dots ,\xi _n). \end{aligned}$$

#### Proof

Use Eqs. (1.25) and (2.19). $\square$

### Conclusion on formal ${\mathcal {T}}$-functions

Here we recalled how Hirota equations are equivalent to Fay identities in the case of formal series times exponentials.

Let us summarize the main points:

- ${\mathcal {T}}$ function here was a formal series of times, multiplied by an exponential factor, that is a single trans-series, or also called a trans-monomial.
- Hirota equations are an infinite set of compatible partial differential equations. We insist that this requires infinitely many variable times: the Tau function has to be defined on an Abelian group $\Gamma$ (which is a group acting on a Grassmannian in the Sato Segal–Wilson formalism), or more generally on an infinite-dimensional space for which times are local coordinates.
- Fay identities are expressed as relations between functions of 2*n* complex variables $(\xi _i, \tilde{\xi }_i)_{i=1,\dots n}$ rather than functions of times. Somehow we have traded an infinite-time dependence to functions of a finite number of complex variables.
  The points $\xi _1,\dots ,\xi _n,\tilde{\xi }_1,\dots ,\tilde{\xi }_n$ are complex variables in which we take residues. Residue is a notion attached to complex variables, i.e., living on a Riemann surface.
- This justifies that we are going to extend the present formalism to the case where $\xi _i, \tilde{\xi }_i$s belong to a Riemann surface, instead of a neighborhood of 0. Fay identities are then functional relations among functions on a Riemann surface at fixed times. This doesn’t need to have a “space” of times, and this does not need series expansions.
- We are thus going to enlarge the context of Fay identities to genuine functions on a Riemann surface, rather than series, trans-monomials or trans-series (trans-series are linear combinations of trans-monomials).
- It is wrong to say that Hirota $\Leftrightarrow$ Fay. Hirota is about formal series, while Fay is about analytic functions. What we have just seen here is that the 2 coincide when the formal series are the Taylor–Laurent expansions of some genuine analytic functions. However, there can exist some formal series (or trans-series) solutions of Hirota, which are not the germs of analytic functions that could be analytically continued to a Riemann surface. Vice versa, there may exist solutions of Fay identities whose singularities could be more complicated than analytic function times exponential of a Laurent polynomial.
  The main concept here is that Hirota is the Taylor expansion (the germ) of Fay.

## Tau functions and Riemann surfaces

### Introduction and motivation

The goal is to exhibit some solutions of Fay identities. This means exhibiting a space $\Gamma$ with some coordinates $\vec {t}$, and some function ${\mathcal {T}}$ on $\Gamma$ satisfying Hirota equations, or equivalently Fay identities.

Fay identities are relations among ${\mathcal {T}}$ evaluated at times Sato—shifted by some $\xi _i$s. Each $\xi _i$ is a complex variable and can be viewed as a local coordinate in a chart of a Riemann surface, so that ${\mathcal {T}}(\vec {t}+ [\xi ])$ is an analytic function on the Riemann surface.

Punctures are defined as the places where ${\mathcal {T}}(\vec {t}+[\xi ])$ is not analytic; this is related to the exponentials of negative powers. This means, by definition, that ${\mathcal {T}}(\vec {t}+[\xi ])$ should be analytic elsewhere, on the whole pointed surface without the punctures. So it is natural to associate a Tau functions to Riemann surfaces. There are several classical ways of associating a space $\Gamma$ to Riemann surfaces and finding a Tau function on it.

**Solutions presented here:**

The challenge would be to find all solutions of Hirota/Fay equations, and at least find some classes of solutions. Without being exhaustive, we focus on 2 classes of solutions here below. Finding a solution means finding a space $\Gamma$, and a function ${\mathcal {T}}$ on it, satisfying the Hirota/Fay equations.

In each case, the “times” are local coordinates or local functions on the space $\Gamma$.

1. **Isospectral Tau functions** (**Section** 5): here we shall consider $\Gamma$ to be the space of meromorphic 1-forms on a fixed Riemann surface ${\Sigma }$. The times are “moments”: if $\Omega \in \Gamma$ is a meromorphic 1-form, the times are $t_{k}(\Omega ) = \underset{0}{\mathop {{\textrm{Res}}}}\ \xi ^k \Omega$. The Sato vector $[\xi ]$ corresponds to a meromorphic 1-form $\Omega _\xi$ having a simple pole at position $\xi$, with residue 1. For a divisor *D*, the Sato vector corresponds to a meromorphic 1-form $\Omega _D$ with simple poles at $\operatorname {supp} D$ with residues = weights. Fay identities are then relations between analytic functions on the Riemann surface. The solutions of Fay identities in this case are known explicitly; they are expressed with Theta functions. This is also called the “reconstruction formula” or “finite-gap solution”, explicit in Section 5.
2. **Isomonodromic Tau functions** (**Section** 6): Here we shall consider $\Gamma$ to be a moduli space of “spectral curves”. A spectral curve is a Riemann surface ${\Sigma }$ equipped with an immersion into a cotangent space ${\Sigma }\hookrightarrow T^*\underline{{\Sigma }}$, $z\mapsto (x,y)$. Times are moments $t_{k} = \underset{0}{\mathop {{\textrm{Res}}}}\ \xi ^k y dx$. A Sato-shifted spectral curve by a Sato vector $[\xi ]$ corresponds to a spectral curve whose immersion *y* has a simple pole at position $\xi$ with residue 1, and more generally for a divisor *D* the Sato-shifted spectral curve by the Sato vector [*D*] corresponds to a spectral curve whose immersion *y* has poles at $\operatorname {supp} D$ with residues = weights.
  The solutions of Fay identities in this case are called “isomonodromic Tau functions” (and they are not the same as the isospectral solutions).
  Small deformations of spectral curves are meromorphic 1-forms $\Omega = \delta (ydx)$, i.e., the tangent space of the moduli space of spectral curve is the space of meromorphic 1-forms. This means that isospectral Tau functions can be recovered as an “infinitesimal” limit of isomonodromic Tau functions.
  This Tau function of spectral curves is developed in Section 6.
3. Many other constructions exist, and we do not claim to be exhaustive here. An example is with $\Gamma$ the deRham space of connections of principal bundles of reductive Lie group *G* over a Riemann surface $\underline{{\Sigma }}$. This is related to Hitchin systems. There is also a construction of Tau function from conformal blocks. These constructions are not presented here.
  In Section 7.1, we present a matrix integral solution of Fay identities.

### Riemann surfaces

Let us first introduce some geometry of Riemann surfaces.

Let $\underline{{\Sigma }}={\mathbb {C}}P^1$, we call it the “base” curve. The base curve may be any Riemann surface but, in this section, for simplicity we restrict to $\underline{{\Sigma }}=$ Riemann sphere = ${\mathbb {C}}P^1$ = ${\mathbb {C}}\cup \{\infty \}$.

#### Definition 4.1

(*Ramified cover*) Let ${\Sigma }$ be a compact Riemann surface of some genus ${\mathfrak {g}}$, equipped with a holomorphic ramified covering map

4.1 $$\begin{aligned} \textrm{x}: {\Sigma }\rightarrow \underline{{\Sigma }}. \end{aligned}$$

#### Definition 4.2

(*Canonical local coordinates*) At each point *p* of ${\Sigma }$, we define $x_p=\textrm{x}(p)$ if $\textrm{x}(p)\ne \infty$ and $x_p=0$ if $\textrm{x}(p)=\infty$. Let

4.2 $$\begin{aligned} a_p := {\operatorname {index}}_p (\textrm{x}-x_p). \end{aligned}$$

If $\textrm{x}(p)\ne \infty$, we have $a_p>0$, and the canonical local coordinate is $\xi _p=(\textrm{x}-x_p)^{\frac{1}{a_p}}$.

If $\textrm{x}(p)=\infty$, we have $a_p<0$, and the canonical local coordinate is $\xi _p=\textrm{x}^{\frac{1}{a_p}} = \textrm{x}^{-\frac{1}{|a_p|}}$.

In both cases,

4.3 $$\begin{aligned} \textrm{x}= x_p+\xi _p^{a_p}. \end{aligned}$$

Remark that if *p* is a ramification point, we have $|a_p|\ge 2$, and thus $\xi _p = (\textrm{x}-x_p)^{1/a_p}$ is ambiguous. It is defined up to a root of unity, or alternatively, there are $|a_p|$ possible different choices for $\xi _p$:

4.4 $$\begin{aligned} \xi _{p,(k)} = \rho _{a_p}^k \xi _p \ , \ k=1,\dots ,|a_p| \ , \qquad \text {where} \ \rho _n = e^{\frac{1}{n}2\pi i}. \end{aligned}$$

#### Definition 4.3

Let

4.5 $$\begin{aligned} \mathfrak M^1({\Sigma }) \end{aligned}$$

be the complex vector space of meromorphic 1-forms on ${\Sigma }$. It is an infinite-dimensional complex vector space.

We associate to a meromorphic 1-form $\Omega$ on ${\Sigma }$ a family of times.

#### Definition 4.4

(*Times of a meromorphic 1-form*) Let $\Omega \in \mathfrak M^1({\Sigma })$ be a meromorphic 1-form on ${\Sigma }$. Since ${\Sigma }$ is compact, $\Omega$ has a finite number of poles.

For a pole *p* of $\Omega$, we can write its polar part as

4.6 $$\begin{aligned} \Omega= &   \sum _{k=0}^{-1+\deg _p\Omega } t_{p,k}(\Omega ) \xi _p^{-k-1}d\xi _p + \text {holomorphic at } p \\= &   \frac{1}{a_p}\sum _{k=0}^{-1+\deg _p\Omega } t_{p,k}(\Omega ) (\textrm{x}-x_p)^{-\frac{k}{a_p}-1}d\textrm{x}+ \text {holomorphic at } p. \end{aligned}$$

The coefficients $t_{p,k}(\Omega )$ of the polar part are worth

4.7 $$\begin{aligned} t_{p,k}(\Omega ) = \mathop {{\textrm{Res}}}_p\, \xi _p^k \ \Omega . \end{aligned}$$

We define the infinite vectors

4.8 $$\begin{aligned} \vec {t}_{p}(\Omega ) := (t_{p,1}(\Omega ),t_{p,2}(\Omega ),\dots ,t_{p,-1+\deg _p\Omega }(\Omega ),0,0,\dots ). \end{aligned}$$

Since $\Omega$ is meromorphic, there can be only a finite number of non-vanishing times.

We write collectively:

4.9 $$\begin{aligned} \textbf{t}(\Omega ) = \{\vec {t}_p(\Omega )\}_{p=\text {poles of }\Omega }. \end{aligned}$$

Remark that if *p* is at the same time a pole and a ramification point, we have several families of times, related by a root of unity:

4.10 $$\begin{aligned} \vec {t}_{p,(k)}(\Omega ) = (\rho _{a_p}^k t_{p,1}(\Omega ), \rho _{a_p}^{2k} t_{p,2}(\Omega ), \dots , \rho _{a_p}^{jk} t_{p,j}(\Omega ), \dots ) := \rho _{a_p}^k(\vec {t}_p(\Omega )). \end{aligned}$$

Remark that if *p* is not a pole of $\Omega$ we have $\vec {t}_p(\Omega )=0$.

#### Monodromies and periods

The curve ${\Sigma }$ has some genus ${\mathfrak {g}}$. It means that there are non-contractible cycles, the fundamental group $\pi _1({\Sigma })$ has rank $2{\mathfrak {g}}$.

##### Definition 4.5

(*Marking*) It is possible (not unique) to find a basis of $2{\mathfrak {g}}$ Jordan loops, $(\mathcal{A}_j,\mathcal{B}_j)_{j=1,\dots ,{\mathfrak {g}}}$ that generate $\pi _1({\Sigma })$, and that have transverse symplectic intersections:

4.11 $$\begin{aligned} \mathcal{A}_i \cap \mathcal{B}_j = \delta _{i,j}, \quad \mathcal{A}_i \cap \mathcal{A}_j = 0, \quad \mathcal{B}_i \cap \mathcal{B}_j = 0, \end{aligned}$$

for all *i*, *j* in $[1,\dots ,{\mathfrak {g}}]$.

##### Remark 4.1

We choose Jordan loops rather than cycles (cycles are homotopy classes of Jordan loops and their linear combinations), because the exact support will matter, since we will integrate meromorphic forms that can have poles.

##### Definition 4.6

(*Periods of a meromorphic 1-form*) Let $\Omega \in \mathfrak M^1({\Sigma })$ be a meromorphic 1-form. Let us assume that the poles of $\Omega$ are not on the support of our marking symplectic Jordan loops. We define the “periods” of $\Omega$ as follows: for all $j\in [1,\dots ,{\mathfrak {g}}]$

4.12 $$\begin{aligned} \epsilon _j(\Omega ) := \frac{1}{2\pi i} \oint _{\mathcal{A}_j} \Omega . \end{aligned}$$

Periods are also called 1st kind times.

##### Remark 4.2

If $\Omega$ would have poles on the support of the Jordan loop, one should replace the integral $\oint _{\mathcal{A}_j}\Omega$ by the regularized integral defined in [36], so that all what follows below remains valid for all $\Omega$. But here, to simplify, let us focus on the case where $\Omega$ has no pole on the marked Jordan loops, and regularized integrals are just usual integrals.

##### Definition 4.7

(*3rd kind times*) For each pole *p* of $\Omega$, let

4.13 $$\begin{aligned} t_{p,0}(\Omega ) = \mathop {{\textrm{Res}}}_p\, \Omega . \end{aligned}$$

#### Canonical decomposition of meromorphic forms

##### Lemma 4.1

(1st kind differentials) For each $i=1,\dots ,{\mathfrak {g}}$, there exists a unique holomorphic 1-form on ${\Sigma }$, denoted $\omega '_{i}$, with no pole, and such that

4.14 $$\begin{aligned} \forall \ j=1,\dots ,{\mathfrak {g}}\ , \qquad \oint _{\mathcal{A}_j} \omega '_{i}=\delta _{i,j}. \end{aligned}$$

$\omega '_1,\dots ,\omega '_{\mathfrak {g}}$ form a basis of $\Omega ^1({\Sigma })$, the space of holomorphic 1-forms on ${\Sigma }$, it is called the basis dual to the marking symplectic Jordan loops.

##### Proof

This is a classical theorem of Riemann surfaces, see [45]. $\square$

##### Lemma 4.2

(3rd kind differentials) Let *p*, *q* be two points on ${\Sigma }$ (we assume that they are not on the support of the marking Jordan loops).

There exists a unique meromorphic 1-form, denoted $\omega '''_{p,q}$, that has a simple pole at *p* of residue $+1$ and a simple pole at *q* of residue $-1$ and no other pole.

4.15 $$\begin{aligned} \mathop {{\textrm{Res}}}_p\, \omega '''_{p,q}= 1 = - \mathop {{\textrm{Res}}}_q \,\omega '''_{p,q}, \end{aligned}$$

and normalized on $\mathcal{A}$-cycles

4.16 $$\begin{aligned} \forall \ i=1,\dots ,{\mathfrak {g}}\ , \qquad \oint _{\mathcal{A}_i} \omega '''_{p,q}=0. \end{aligned}$$

If $D=\sum _{i=1}^\ell \alpha _i.[z_i]$ is a divisor of degree $\deg D=0$, we define

4.17 $$\begin{aligned} \omega '''_D := \sum _{i=1}^\ell \alpha _i \omega '''_{z_i,o}, \end{aligned}$$

where *o* is a generic point of ${\Sigma }$, and in fact $\omega '''_D$ is independent of it. Remark that $\omega '''_{p,q} = \omega '''_{[p]-[q]}$.

$\omega '''_D$ is the unique meromorphic 1-form that has simple poles at the support of *D* with residues = weights and no other poles, and vanishing $\mathcal{A}$-cycle integrals.

##### Proof

This is a classical theorem, see [45]. $\square$

##### Lemma 4.3

(2nd kind differentials) Let *p* be a point on ${\Sigma }$, and $k\ge 1$ (we assume that *p* is not on the support of the marking Jordan loops).

There exists a unique meromorphic 1-form, denoted $\omega ''_{p,k}$, that has a pole at *p* of degree $k+1$ and no other pole, and behaving like

4.18 $$\begin{aligned} \omega ''_{p,k} = \xi _p^{-k-1}d\xi _{p} + {\text {holomorphic at }} p, \end{aligned}$$

and normalized on $\mathcal{A}$-cycles

4.19 $$\begin{aligned} \forall \ i=1,\dots ,{\mathfrak {g}}\ , \qquad \oint _{\mathcal{A}_i} \omega ''_{p,k}=0. \end{aligned}$$

##### Proof

This is a classical theorem, see [45] $\square$

##### Lemma 4.4

(Decomposition) Every meromorphic 1-form $\Omega$ can be uniquely written

4.20 $$\begin{aligned} \Omega= &   \sum _{p=\text {poles of }\Omega } \sum _{k=1}^{-1+\deg _p\Omega } t_{p,k}(\Omega ) \ \omega ''_{p,k} \\  &   + \sum _{p=\text {poles of }\Omega } t_{p,0}(\Omega ) \ \omega '''_{p,o} + 2\pi \textrm{i}\,\sum _{i=1}^{{\mathfrak {g}}} \epsilon _i(\Omega ) \ \omega '_i. \end{aligned}$$

(here *o* is an arbitrary generic point of ${\Sigma }$, and the decomposition is actually independent of it).

In other words, the times $\textbf{t}(\Omega )$ are the coordinates of $\Omega$ in the canonical basis of forms.

##### Proof

This is a classical theorem, see [45]. The main idea is that the difference between $\Omega$ and the right-hand side is a 1-form without poles, and with vanishing $\mathcal{A}_i$ integrals, so it must vanish. $\square$

### More geometry of Riemann surfaces

Before showing explicit solutions of the Fay/Hirota equations, we first need to introduce more geometry, in particular, Theta functions and Fay’s Prime form [45, 46, 72].

#### Holomorphic forms, Riemann periods, Theta

##### Proposition 4.1

(Riemann) Define the Riemann period matrix using the normalized holomorphic 1-forms of Lemma 4.1

4.21 $$\begin{aligned} \tau _{i,j} = \oint _{\mathcal{B}_i} \omega '_j. \end{aligned}$$

$\tau$ is a ${\mathfrak {g}}\times {\mathfrak {g}}$ matrix, belonging to the Siegel space, which means it is symmetric and its imaginary part is positive definite:

4.22 $$\begin{aligned} \tau =\tau ^t, \quad \Im \tau >0. \end{aligned}$$

##### Proof

This is a very classical theorem going back to Riemann [45]. $\square$

##### Remark 4.3

Although every Riemann period matrix is in the Siegel space, the converse is not true, not all matrices in the Siegel space are Riemann periods of Riemann surfaces. The subset of the Siegel space that are periods of Riemann surfaces is characterized by the Krichever–Novikov conjecture, later proved by T. Shiota [11], and the proof is closely related to the isospectral solution of Hirota equations presented below.

##### Definition 4.8

(*Abel-Jacobi map*) Let *o* be a generic point of ${\Sigma }$ fixed once for all, called the origin. We define the Abel–Jacobi map:

4.23 $$\begin{aligned} {\Sigma }^{\text {universal cover}}\rightarrow &   {\mathbb {C}}^{\mathfrak {g}}\\z\mapsto &   \mathfrak a(z) = (\mathfrak a_1(z),\dots ,\mathfrak a_{\mathfrak {g}}(z)) \quad \text {where } \ \mathfrak a_i(z)= \int _o^z \omega '_i. \end{aligned}$$

They have monodromies

4.24 $$\begin{aligned} \mathfrak a_i(z+\mathcal{A}_j) = \mathfrak a_i(z) + \delta _{i,j}, \end{aligned}$$

4.25 $$\begin{aligned} \mathfrak a_i(z+\mathcal{B}_j) = \mathfrak a_i(z) + \tau _{i,j}, \end{aligned}$$

and therefore

4.26 $$\begin{aligned} {\Sigma }\rightarrow &   \mathbb J = {\mathbb {C}}^{\mathfrak {g}}/({\mathbb {Z}}^{\mathfrak {g}}+\tau {\mathbb {Z}}^{\mathfrak {g}}), \\z\mapsto &   \mathfrak a(z) \ \mod {\mathbb {Z}}^{\mathfrak {g}}+\tau {\mathbb {Z}}^{\mathfrak {g}}\end{aligned}$$

is well defined. $\mathbb J = {\mathbb {C}}^{\mathfrak {g}}/({\mathbb {Z}}^{\mathfrak {g}}+\tau {\mathbb {Z}}^{\mathfrak {g}})$ is called the Jacobian of ${\Sigma }$.

If $D=\sum _{i=1}^\ell \alpha _i.z_i$ is a divisor of points of ${\Sigma }$, we define by linearity

4.27 $$\begin{aligned} \mathfrak a(D) = \sum _{i=1}^\ell \alpha _i \mathfrak a(z_i). \end{aligned}$$

If $\deg D=0$, $\mathfrak a(D)$ is independent of the origin *o*.

##### Definition 4.9

(*Riemann Theta function*) Let $\tau$ be a Siegel matrix. We define the function

4.28 $$\begin{aligned} {\mathbb {C}}^{\mathfrak {g}}\rightarrow &   {\mathbb {C}}\\u=(u_1,\dots ,u_{\mathfrak {g}})\mapsto &   \Theta (u) = \Theta (u;\tau ) =: \sum _{\textbf{n}\in {\mathbb {Z}}^{\mathfrak {g}}} e^{2\pi \textrm{i}\,(u,\textbf{n})} \ e^{\pi \textrm{i}\,(\textbf{n},\tau \textbf{n})} \end{aligned}$$

(very often we shall consider $\tau$ fixed and view $\Theta$ as only a function of $u\in {\mathbb {C}}^{\mathfrak {g}}$). Thanks to $\Im \tau >0$, the series is absolutely convergent for all $u\in {\mathbb {C}}^{\mathfrak {g}}$ and is an analytic entire function on ${\mathbb {C}}^{\mathfrak {g}}$.

##### Proposition 4.2

(Riemann) $\Theta$ obeys the following properties:

4.29 $$\begin{aligned} \Theta (-u)= &   \Theta (u) \\\Theta (u+\textbf{n})= &   \Theta (u) \\\Theta (u+\tau \textbf{n})= &   \Theta (u) \ e^{-2\pi \textrm{i}\,(u,\textbf{n})} e^{-\pi \textrm{i}\,(\textbf{n},\tau \textbf{n})} \\\Theta \left( u+\frac{1}{2}\textbf{n}+\frac{1}{2} \tau \textbf{m}\right)= &   0 \qquad \text {iff} \ (\textbf{n},\textbf{m})\in 2{\mathbb {Z}}+1 \end{aligned}$$

#### Fay’s prime forms

$\Theta$ functions can be defined for any Siegel matrix $\tau$. From now on, we shall specialize to $\tau$ the Riemann period matrix of ${\Sigma }$.

##### Definition 4.10

(*Half-integer characteristic*) Let $\textbf{n},\textbf{m}\in {\mathbb {Z}}^{\mathfrak {g}}$.

4.30 $$\begin{aligned} \chi := \frac{1}{2}\textbf{n}+\frac{1}{2} \tau \textbf{m}\end{aligned}$$

is called a half-integer characteristics.

It is called odd iff $(\textbf{n},\textbf{m})\in 2{\mathbb {Z}}+1$.

It is called non-singular iff the vector $\Theta '(\chi ) = (\Theta '_1(\chi ),\dots ,\Theta '_{\mathfrak {g}}(\chi ))\ne 0$.

It is known that there always exists some non-singular odd half-integer characteristic.

From now on, let us choose one of them, fixed once for all. Let us define the following holomorphic 1-form

4.31 $$\begin{aligned} h_\chi := \sum _{i=1}^{\mathfrak {g}}\Theta '_i(\chi ) \omega _i. \end{aligned}$$

It is well known that $h_\chi$ is a holomorphic 1-form, that has ${\mathfrak {g}}-1$ double zeros on ${\Sigma }$.

##### Definition 4.11

(*Fay’s prime form*) Let

4.32 $$\begin{aligned} E(z_1,z_2) := \frac{\Theta (\mathfrak a(z_1)-\mathfrak a(z_2)+ \chi )}{\sqrt{h_\chi (z_1) \ h_\chi (z_2)}}. \end{aligned}$$

If $D =\sum _{i=1}^\ell \alpha _i.z_i$ is a divisor, we define

4.33 $$\begin{aligned} E(D) := \prod _{i<j} E(z_i,z_j)^{-\alpha _i\alpha _j}. \end{aligned}$$

Remark:

4.34 $$\begin{aligned} E(z_1,z_2) = E([z_1]-[z_2]). \end{aligned}$$

##### Proposition 4.3

(Fay) $E(z_1,z_2)$ is locally analytic of $z_1$ (resp. $z_2$), or more precisely is analytic on the universal cover. In particular, it has no poles (the ${\mathfrak {g}}-1$ doubles zeros of $h_\chi$ happen to be exactly ${\mathfrak {g}}-1$ zeros of $\Theta$ in the numerator). It vanishes only at $z_1=z_2$ and vanishes linearly. In any local coordinate $\xi _i=\xi (z_i)$, it behaves as

4.35 $$\begin{aligned} E(z_1,z_2) \mathop {\sim }_{z_1\rightarrow z_2} \frac{\xi _1-\xi _2}{\sqrt{d\xi _1 d\xi _2}} \ (1+O(\xi _1-\xi _2)). \end{aligned}$$

##### Definition 4.12

(*Szegö kernel*) Let $\Omega$ a meromorphic 1-form on ${\Sigma }$. Let

4.36 $$\begin{aligned} \zeta (\Omega )=(\zeta _1(\Omega ),\dots ,\zeta _{\mathfrak {g}}(\Omega )) := \frac{1}{2\pi \textrm{i}\,}\oint _{\mathcal{B}-\tau \mathcal{A}} \Omega , \end{aligned}$$

4.37 $$\begin{aligned} \text {i.e.} \qquad \zeta _{i}(\Omega ) = \frac{1}{2\pi \textrm{i}\,} \left( \oint _{\mathcal{B}_i}\Omega - \sum _{j=1}^{\mathfrak {g}}\tau _{i,j} \oint _{\mathcal{A}_j} \Omega \right) . \end{aligned}$$

Let the Szegö kernel

4.38 $$\begin{aligned} \psi ([z_1]-[z_2];\Omega ) := \frac{\Theta (\zeta (\Omega )+\mathfrak a(z_1)-\mathfrak a(z_2)+\chi )}{E(z_1,z_2)\Theta (\zeta (\Omega )+\chi ) } \ e^{\int _{z_2}^{z_1} \Omega }\ e^{-2\pi \textrm{i}\,(\epsilon (\Omega ),\mathfrak a(z_1)-\mathfrak a(z_2))}. \end{aligned}$$

More generally, for a divisor *D* of degree 0:

4.39 $$\begin{aligned} \psi (D;\Omega ) = \frac{\Theta (\zeta (\Omega )+\mathfrak a(D)+\chi )}{E(D) \ \Theta (\zeta (\Omega )+\chi )} e^{\sum _{i=1}^\ell \alpha _i\int _{o}^{z_i} \Omega } e^{-2\pi \textrm{i}\,(\epsilon (\Omega ),\mathfrak a(D))}. \end{aligned}$$

##### Lemma 4.5

If $\Omega$ has poles with integer residues, then the Szegö kernel has no monodromies:

4.40 $$\begin{aligned} \psi ([z_1+\gamma ]-[z_2];\Omega ) = \psi ([z_1]-[z_2];\Omega ) = \psi ([z_1]-[z_2+\gamma ];\Omega ) \end{aligned}$$

for all cycle $\gamma$.

##### Proof

$\bullet$ If $\gamma =\sum _{i=1}^{\mathfrak {g}}n_i \mathcal{A}_i$ we have

4.41 $$\begin{aligned} \mathfrak a(z_1+\gamma ) = \mathfrak a(z_1)+ \textbf{n}\qquad \text { where }\textbf{n}=(n_1,\dots ,n_{\mathfrak {g}}), \end{aligned}$$

and $\Theta (u+\textbf{n})=\Theta (u)$, this implies

4.42 $$\begin{aligned} \psi ([z_1+\gamma ]-[z_2];\Omega )= &   \psi ([z_1]-[z_2];\Omega ) \ e^{\int _\gamma \Omega } \ e^{-2\pi \textrm{i}\,(\epsilon (\Omega ),\textbf{n})} \\= &   \psi ([z_1]-[z_2];\Omega ), \end{aligned}$$

because

4.43 $$\begin{aligned} \int _\gamma \Omega = \sum _{i=1}^{\mathfrak {g}}n_i \oint _{\mathcal{A}_i} \Omega = 2\pi \textrm{i}\,\sum _{i=1}^{\mathfrak {g}}n_i \epsilon _i(\Omega ) = 2\pi \textrm{i}\,(\epsilon (\Omega ),\textbf{n}). \end{aligned}$$

$\bullet$ If $\gamma =\sum _{i=1}^{\mathfrak {g}}n_i \mathcal{B}_i$, we have

4.44 $$\begin{aligned} \mathfrak a(z_1+\gamma ) = \mathfrak a(z_1)+ \tau \textbf{n}\qquad \text { where }\textbf{n}=(n_1,\dots ,n_{\mathfrak {g}}), \end{aligned}$$

and $\Theta (u+\tau \textbf{n})=\Theta (u) e^{-2\pi \textrm{i}\,((u,\textbf{n}) + \frac{1}{2} (\textbf{n},\tau \textbf{n}) ) }$, this implies

4.45 $$\begin{aligned} \psi ([z_1+\gamma ]-[z_2];\Omega )= &   \psi ([z_1]-[z_2];\Omega ) \ \frac{e^{-2\pi \textrm{i}\,((\zeta (\Omega )+\mathfrak {a}(D)+\chi ,\textbf{n}) + \frac{1}{2} (\textbf{n},\tau \textbf{n}) )}}{e^{-2\pi \textrm{i}\,((\mathfrak {a}(D)+\chi ,\textbf{n}) + \frac{1}{2} (\textbf{n},\tau \textbf{n}) )}} \nonumber \\  &   e^{\int _\gamma \Omega } \ e^{-2\pi \textrm{i}\,(\epsilon (\Omega ),\tau \textbf{n})} \nonumber \\= &   \psi ([z_1]-[z_2];\Omega ) \ e^{-2\pi \textrm{i}\,(\zeta (\Omega ),\textbf{n})} \ e^{\int _\gamma \Omega } \ e^{-2\pi \textrm{i}\,(\epsilon (\Omega ),\tau \textbf{n})} \\= &   \psi ([z_1]-[z_2];\Omega ), \end{aligned}$$

because

4.46 $$\begin{aligned} \int _\gamma \Omega = \sum _{i=1}^{\mathfrak {g}}n_i \oint _{\mathcal{B}_i} \Omega = 2\pi \textrm{i}\,(\zeta (\Omega )+\tau \epsilon (\Omega ),\textbf{n}). \end{aligned}$$

$\bullet$ If $\gamma$ is a small circle around a pole *p* of $\Omega$ with residue $\mathop {{\textrm{Res}}}_p\, \Omega = t_{p,0}(\Omega )$, we have

4.47 $$\begin{aligned} \psi ([z_1+\gamma ]-[z_2];\Omega )= &   \psi ([z_1]-[z_2];\Omega ) \ e^{2\pi \textrm{i}\,t_{p,0}(\Omega )} \\= &   \psi ([z_1]-[z_2];\Omega ), \end{aligned}$$

because we assumed $t_{p,0}(\Omega )\in \mathbb Z$. $\square$

## 1st construction: $\Gamma =$ space of meromorphic forms

Here we present the so-called Theta-function solution of Fay identities, also called the finite-gap Tau function or Isospectral Tau function, or reconstruction formula.

Let ${\Sigma }$ a compact Riemann surface of genus ${\mathfrak {g}}$, equipped with a Jordan loops marking, and with a degree *d* covering map $\textrm{x}:{\Sigma }\rightarrow \underline{{\Sigma }}$.

The Abelian space $\Gamma$ considered in this 1st construction is the vector space of meromorphic 1-forms on ${\Sigma }$, modulo 1st kind and 3rd kind forms.

### Definition 5.1

Let $\mathcal P$ be a finite set of points of ${\Sigma }$, called the “poles” (chosen out of the support of Jordan loops markings). Let

5.1 $$\begin{aligned} \mathfrak M^1_\mathcal {P}({\Sigma }) := \{ \Omega \in \mathfrak M^1({\Sigma }) \ | \ \Omega \text { has poles only at } \mathcal P \text { or no pole}\}. \end{aligned}$$

5.2 $$\begin{aligned} \mathfrak M^1_\mathcal {P}({\Sigma })''' := \{ \Omega \in \mathfrak M^1({\Sigma }) \ | \ \Omega \text { has only simple poles at } \mathcal P \text { or no pole}\}. \end{aligned}$$

5.3 $$\begin{aligned} \Gamma _\mathcal {P} := \mathfrak M^1_\mathcal {P}({\Sigma })/\mathfrak M^1_\mathcal {P}({\Sigma })'''. \end{aligned}$$

With the time map on $\Gamma _\mathcal {P}$:

5.4 $$\begin{aligned} \Omega\mapsto &   \{t_{p,k}(\Omega )\}_{p\in \mathcal P, \ k\ge 1}. \end{aligned}$$

For any $\Omega$, only finitely many times are nonzero. Times are coordinates on $\Gamma _\mathcal {P}$.

### Proposition 5.1

(Preimage of the Sato vector) Let $p\in \mathcal P$. Let $U_{p}\subset {\Sigma }$ be an open simply connected neighborhood of *p* in which the canonical local coordinate $\xi _{p}$ is defined.

Let $z\in {\Sigma }$. If $z\in U_{p}$, let $\xi =\xi _{p}(z)$.

The 3rd kind differential of Lemma 4.2, $\omega '''_{z,p}$ is the preimage of the Sato vector $[\xi _p(z)]$. Indeed as a formal series of $\xi$, we have

5.5 $$\begin{aligned} \vec {t}_p(\omega '''_{z,p}) = [\xi ] = [\xi _p(z)] . \end{aligned}$$

If $D=\sum _{i=1}^\ell \alpha _i.z_i$ is a divisor whose points are in $U_p$, we have, as a formal series of $\xi _p(z_i)s$, the time map of the 3rd kind differential $\omega '''_D$ is the Sato vector [*D*]:

5.6 $$\begin{aligned} \vec {t}_p\left( \omega '''_D \right) = \vec {t}_p\left( \sum _{i=1}^\ell \alpha _i \omega '''_{z_i,p}\right) = [D] . \end{aligned}$$

### Proof

Let $z\in U_p$, and consider the 1-form $\omega '''_{z,p}(\tilde{z})$, it behaves as

5.7 $$\begin{aligned} \omega '''_{z,p}(\tilde{z}) \sim \frac{d\xi _p(\tilde{z})}{\xi _p(\tilde{z})-\xi _p(z)} - \frac{d\xi _p(\tilde{z})}{\xi _p(\tilde{z})} + \text {analytic}, \end{aligned}$$

and notice that the times will not depend on the analytic part.

If *z* is close to *p*, so that $|\xi _p(z)|<|\xi _p(\tilde{z})|$ we have the Taylor expansion in ${\mathbb {C}}[[\xi _p(z)]]$:

5.8 $$\begin{aligned} \omega '''_{z,p}(\tilde{z}) \sim \frac{d\xi _p(\tilde{z})}{\xi _p(\tilde{z})-\xi _p(z)} - \frac{d\xi _p(\tilde{z})}{\xi _p(\tilde{z})} \sim \sum _{k=1}^\infty \frac{\xi _p(z)^{k}}{\xi _p(\tilde{z})^{k+1}} \ d\xi _p(\tilde{z}) . \end{aligned}$$

This implies that, in the sense of formal series in ${\mathbb {C}}[[\xi _p(z)]]$, we have

5.9 $$\begin{aligned} t_{p,k}(\omega '''_{z,p}) = \xi _p(z)^{k}, \end{aligned}$$

which shows that this is indeed the Sato vector. $\square$

### Remark 5.1

It is important to notice that $\omega '''_{z,p}(\tilde{z})$ is a well-defined meromorphic 1-form defined on $\tilde{z} \in {\Sigma }$, whereas the formal germ $\sum _{k=1}^\infty \frac{\xi _p(z)^{k}}{\xi _p(\tilde{z})^{k+1}} \ d\xi _p(\tilde{z})$ makes sense only in a neighborhood of *p*, it is the Taylor series expansion in the neighborhood.

In other words, $\Omega =\omega '''_{z,p}$ is defined globally on ${\Sigma }$, whereas the writing as a Sato vector is a Taylor series germ only within a neighborhood.

### Remark 5.2

The time map is invertible on the quotient by holomorphic and 3rd kind 1-forms. Somehow in the map, the times “forget” the period and 3rd kind coordinates.

Usually in integrable systems, one wants to find a Tau function that is a function (or formal series) of times. We shall rather consider a Tau function that depends on a 1-form $\Omega \in \mathfrak M^1({\Sigma })$:

### Definition 5.2

(*Hirota - Fay equations*) A function

5.10 $$\begin{aligned} {\mathcal {T}}: \mathfrak M^1({\Sigma }) \rightarrow {\mathbb {C}}\end{aligned}$$

is said to satisfy Fay identities, iff:

5.11 $$\begin{aligned} \forall \ z_1,z_2,z_3,z_4 \in {\Sigma }  &   \\{\mathcal {T}}(\Omega ){\mathcal {T}}(\Omega +\omega '''_{z_1,z_2}+\omega '''_{z_3,z_4})= &   {\mathcal {T}}(\Omega +\omega '''_{z_1,z_2}){\mathcal {T}}(\Omega +\omega '''_{z_3,z_4}) \\  &   - {\mathcal {T}}(\Omega +\omega '''_{z_1,z_4}){\mathcal {T}}(\Omega +\omega '''_{z_3,z_2}). \end{aligned}$$

In other words, ${\mathcal {T}}$ is a section of a line bundle $\mathcal L \rightarrow \mathfrak M^1({\Sigma })$ that descends to a Tau function on $\Gamma _\mathcal {P}$ in the times coordinates

5.12 $$\begin{aligned}&\mathcal L&\\&\downarrow&\ \uparrow {\mathcal {T}}= \text {section} \\&\mathfrak M^1({\Sigma })&\\&\downarrow&\textbf{t}=\{\vec {t}_p\}_{p\in \mathcal P} \\&\Gamma _\mathcal {P}&\end{aligned}$$

Equation (5.11) has been solved by the so-called reconstruction formula [25, 63–67], using theta functions which we recall now.

### Theta Tau function

This 1st way of obtaining solutions to Hirota/Fay equations is sometimes called “finite-gap solution” or “isospectral Tau function”, or the “reconstruction formula”. It is mostly the work of the Russian school, particularly developed by Its, Matveev, Krichever, Novikov, Dubrovin, and many others [26, 52, 63, 64]. We refer also to the textbook [7].

#### Theorem 5.1

(Fay’s Theorem “Theta functions satisfy Fay identities”) let $D=\sum _{i=1}^n [z_i]-[\tilde{z}_i]$ be a supersymmetric divisor. The Sezgö kernel satisfy the determinantal formula

5.13 $$\begin{aligned} \psi (D;\Omega ) = \det _{i,j=1,\dots ,n} \psi ([z_i]-[\tilde{z}_j];\Omega ). \end{aligned}$$

#### Proof

Due to Fay [46]. Sketching the idea of Fay’s proof is that the ratio of both sides, viewed as a function of any point of the support of *D*, has no monodromy and no pole. Therefore, it must be a constant, independent of the points of *D*. It suffices to evaluate it in a limit of coinciding points, and in this limit the ratio tends to 1. Notice that in genus zero Eq. (5.13) reduces to the Cauchy determinant formula. $\square$

As an immediate corollary, we obtain the famous “reconstruction formula” for Tau functions (see textbook [7]):

#### Theorem 5.2

(Theta Tau function) Let

5.14 $$\begin{aligned} {\mathcal {T}}(\Omega ) = \Theta (\zeta (\Omega )+\chi ) \ e^{\frac{1}{2} Q(\Omega ,\Omega )} \ e^{-2\pi \textrm{i}\,(\epsilon (\Omega ),\zeta (\Omega ))} \ e^{-\pi \textrm{i}\,(\epsilon (\Omega ),\tau \epsilon (\Omega ))}, \end{aligned}$$

where *Q* is the quadratic form

5.15 $$\begin{aligned} Q(\Omega ,\Omega )= &   \sum _{p\in \mathcal P} \sum _{k=1}^{-1+\deg _p\Omega } \frac{1}{k} t_{p,k}(\Omega ) \mathop {{\textrm{Res}}}_p\, \xi _p^{-k}\Omega \\  &   +\sum _{p\in \mathcal P} t_{p,0}(\Omega ) \int _o^p \Omega + \sum _{i=1}^{\mathfrak {g}}\epsilon _i(\Omega ) \oint _{\mathcal{B}_i} \Omega . \end{aligned}$$

It is such that

5.16 $$\begin{aligned} \frac{{\mathcal {T}}(\Omega +\omega '''_D)}{{\mathcal {T}}(\Omega )} = \psi (D;\Omega ). \end{aligned}$$

And, viewed as a function of the times $\textbf{t}(\Omega )$, ${\mathcal {T}}(\Omega )$ satisfies the Fay determinantal formulas of Definition 5.2, and therefore, it satisfies Hirota equations for each family of times $\vec {t}_p(\Omega )$ associated with each $p\in \mathcal P$.

#### Proof

An easy computation yields

5.17 $$\begin{aligned} \zeta (\Omega +\omega '''_D) = \zeta (\Omega ) + \mathfrak a(D). \end{aligned}$$

Let us define

5.18 $$\begin{aligned} \tilde{Q}(\Omega ,\Omega )= &   Q(\Omega -2\pi \textrm{i}\,(\epsilon (\Omega ),\omega '),\Omega -2\pi \textrm{i}\,(\epsilon (\Omega ),\omega ')) \\= &   Q(\Omega ,\Omega ) - 4\pi \textrm{i}\,(\epsilon (\Omega ),\zeta (\Omega )) - 2\pi \textrm{i}\,(\epsilon (\Omega ),\tau \epsilon (\Omega )), \end{aligned}$$

this shows that

5.19 $$\begin{aligned} {\mathcal {T}}(\Omega ) = \Theta (\zeta (\Omega )+\chi ) \ e^{\tilde{Q}(\Omega ,\Omega )}. \end{aligned}$$

We have

5.20 $$\begin{aligned} \tilde{Q}(\Omega +\omega '''_D,\Omega +\omega '''_D) = \tilde{Q}(\Omega ,\Omega ) + 2\sum _{i=1}^\ell \alpha _i \int _o^{z_i} \Omega -2\ln {E(D)}, \end{aligned}$$

this gives

5.21 $$\begin{aligned} \frac{{\mathcal {T}}(\Omega +\omega '''_D)}{{\mathcal {T}}(\Omega )} = \psi (D;\Omega ). \end{aligned}$$

Then, Theorem 5.1 implies that ${\mathcal {T}}$ is a Tau function solution to Fay identities and thus Hirota equations. $\square$

## 2nd construction: spectral curves

We shall now present another distinct solution of Fay identities.

Let the base curve $\underline{{\Sigma }}$ be a compact Riemann surface, we don’t necessarily choose $\underline{{\Sigma }}={\mathbb {C}}P^1$.

### Spectral curves

Here we take the following definition^1^:

#### Definition 6.1

(*Spectral curve*) A spectral curve is a meromorphic Lagrangian immersion of a compact Riemann surface ${\Sigma }$ of some genus ${\mathfrak {g}}$ into the total cotangent bundle $T^*\underline{{\Sigma }}$ of the base curve $\underline{{\Sigma }}$. Let $\textrm{i}\,=(\textrm{x},\textrm{y})$ the immersion $\textrm{i}\,:{\Sigma }\hookrightarrow T^*\underline{{\Sigma }}$, where $\textrm{x}$ is the projection to the base, and $\textrm{y}$ the value in the fiber ($\textrm{y}$ is thus the restriction of the tautological 1-form of $T^*\underline{{\Sigma }}$ to the image of the curve, $\textrm{y}$ is called the Liouville form):6.1The base projection $\textrm{x}:{\Sigma }\rightarrow \underline{{\Sigma }}$ is a holomorphic ramified covering map. $\textrm{y}$ is a meromorphic 1-form on ${\Sigma }$:

6.2 $$\begin{aligned} \textrm{y}\in \mathfrak M^1({\Sigma }). \end{aligned}$$

Useful remark: when $\underline{{\Sigma }}={\mathbb {C}}P^1$, we can use the 1-form $d\textrm{x}$ to identify the fiber of $T^*{\mathbb {C}}P^1$ with ${\mathbb {C}}$, i.e., write the 1-form $\textrm{y}$ as

6.3 $$\begin{aligned} \textrm{y}= yd\textrm{x}, \ \ \text {fiber of } T^*{\mathbb {C}}P^1 \sim {\mathbb {C}}. \end{aligned}$$

That is, we can replace the meromorphic 1-form $\textrm{y}$ by a meromorphic function *y*.

#### Definition 6.2

(*Times of a spectral curve*) The times of a spectral curve ${\mathcal {S}}$ are defined as the times of the 1-form $\textrm{y}$ ($\textrm{y}=y d\textrm{x}$ in the case $\underline{{\Sigma }}={\mathbb {C}}P^1$). Let $\mathcal P=\{ \text {poles of }\textrm{y}\}$, these are called the punctures

6.4 $$\begin{aligned} \mathcal P = \{\text {punctures}\} = \{ \text {poles of }\textrm{y}\}. \end{aligned}$$

6.5 $$\begin{aligned} \textbf{t}= \textbf{t}({\mathcal {S}}) = \{t_{p,k}\}_{p\in \mathcal P, \ k\ge 1 } \ , \qquad t_{p,k} = t_{p,k}({\mathcal {S}}) = t_{p,k}(\textrm{y}) = \mathop {{\textrm{Res}}}_{p}\, \xi _p^k \textrm{y}. \end{aligned}$$

Since $\textrm{y}$ is meromorphic, there are only finitely many punctures and finitely many non-vanishing times.

#### Definition 6.3

(*Periods of the spectral curve*) We define the “periods” for all $j\in [1,\dots ,{\mathfrak {g}}]$.

6.6 $$\begin{aligned} \epsilon _j =\epsilon _j({\mathcal {S}}) = \epsilon _j(\textrm{y}) := \frac{1}{2\pi i} \oint _{\mathcal{A}_j} \textrm{y}\qquad \left( = \frac{1}{2\pi i} \oint _{\mathcal{A}_j} y d\textrm{x}\right) , \end{aligned}$$

where we wrote the last expression in the case $\underline{{\Sigma }}={\mathbb {C}}P^1$.

Periods associated with punctures are called **3rd kind periods** or **3rd kind times**, and are worth:

6.7 $$\begin{aligned} t_{p,0} = t_{p,0}({\mathcal {S}}) = t_{p,0}(\textrm{y}) := \mathop {{\textrm{Res}}}_{p}\, \textrm{y}\qquad \left( = \mathop {{\textrm{Res}}}_p \,y d\textrm{x}\right) . \end{aligned}$$

As we shall see these periods of 1st and 3rd kind are associated with monodromic datas, they won’t obey isomonodromic deformation equations.

#### Proposition 6.1

(Sato shift) Let $D =\sum _{i=1}^{\ell } \alpha '_i.z_i$ be a divisor of points of ${\Sigma }$ (whose support is assumed disjoint from the punctures, marked Jordan loops and ramification or nodal points).

There exists $r>0$ and there exists a 1-parameter family of spectral curves ${\mathcal {S}}_{u}$ for $u\in [0,r[$, with immersions $(\textrm{x}_u,\textrm{y}_u):{\Sigma }_u\hookrightarrow T^*\underline{{\Sigma }}$, such that:

$\bullet$ $\forall u\in [0,r[$, the punctures $\mathcal P_u$ (poles of $\textrm{y}_u$) are in holomorphic bijection with those of $\mathcal P$, with the same $\textrm{x}$ projection and with same degrees, i.e., there is a continuous family of bijections $p\mapsto p_u$ for each *u*, such that

6.8 $$\begin{aligned} \textrm{x}_u(p_u) = \textrm{x}(p),\quad \deg _{p_u} \textrm{y}_u = \deg _p \textrm{y},\quad p_{u=0}=p. \end{aligned}$$

$\bullet$ $\forall u\in [0,r[$ there exists a continuous family of divisor $D_u = \sum _{i=1}^\ell \alpha '_i.\zeta _u(z_i)$ of ${\Sigma }_u$ such that

6.9 $$\begin{aligned} \forall i = 1,\dots , \ell \qquad \textrm{x}_u(\zeta _u(z_i)) = \textrm{x}(z_i), \qquad \ \ \zeta _{u=0}(z_i)=z_i. \end{aligned}$$

$\bullet$ ${\Sigma }_u{\setminus } (\mathcal P_u \cup \operatorname {supp} D_u)$ is isotopic to ${\Sigma }{\setminus } (\mathcal P \cup \operatorname {supp} D)$,

$\bullet$ and in fibers of $T^*\underline{{\Sigma }}$

6.10 $$\begin{aligned} \forall u\in [0,r[ \quad \frac{d}{d u} \textrm{y}_u = \omega '''_{D_u}. \end{aligned}$$

Its times are worth

6.11 $$\begin{aligned} t_{p,k}({\mathcal {S}}_u) = t_{p,k}({\mathcal {S}}) + \delta _{k,0} \sum _{i=1}^\ell \alpha '_i (\delta _{p,\zeta _u(z_i)} -\delta _{p,\infty }), \end{aligned}$$

and with constant periods

6.12 $$\begin{aligned} \epsilon _i({\mathcal {S}}_u) = \epsilon _i({\mathcal {S}}). \end{aligned}$$

We denote it:

6.13 $$\begin{aligned} {\mathcal {S}}_u = {\mathcal {S}}+u[D]. \end{aligned}$$

We admit this proposition here without proof, we refer to [37, 39]. We give an illustrative example below in Section 6.1.1.

#### Lemma 6.1

(Associativity of the shift) The Sato addition is associative and commutative.

If *D* and $D'$ are two supersymmetric divisors, we have

6.14 $$\begin{aligned} {\mathcal {S}}+u(D+D') = ({\mathcal {S}}+ u D) + u D'_u = ({\mathcal {S}}+ u D') + u D_u. \end{aligned}$$

If $D=\sum _{i=1}^\ell \alpha _i z_i$ we have

6.15 $$\begin{aligned} \vec {t}_p({\mathcal {S}}+ u D) = \vec {t}_p({\mathcal {S}}) + u \sum _{i=1}^\ell \alpha _i [\xi _p(z_i)]. \end{aligned}$$

Instead of giving the proof of the proposition and lemma, let us show how it works on an example:

#### Prototypical example

Take $\underline{{\Sigma }}={\mathbb {C}}P^1$ and ${\Sigma }={\mathbb {C}}P^1$ and consider the spectral curve ${\mathcal {S}}$ (called Airy Spectral curve) defined by the immersion

6.16 $$\begin{aligned} {\mathcal {S}}: \left\{ \begin{array}{lll} \textrm{x}(z) &  = &  z^2 \\ \textrm{y}(z) &  = &  2 z^2 dz \end{array} \right. \end{aligned}$$

or in other words with $\textrm{y}=y d\textrm{x}$, and $d\textrm{x}=2z dz$, we have

6.17 $$\begin{aligned} y(z) = z. \end{aligned}$$

It satisfies the algebraic equation:

6.18 $$\begin{aligned} y^2-x=0. \end{aligned}$$

There is a unique puncture $\mathcal P=\{\infty \}$, and its only non-vanishing time is:

6.19 $$\begin{aligned} t_{\infty ,3}({\mathcal {S}}) = -2. \end{aligned}$$

Let $z_1\in {\Sigma }$, and $X_1 = \textrm{x}(z_1)=z_1^2 \in \underline{{\Sigma }}$. Let us define the function $\zeta _u(z_1)$ as the solution of

6.20 $$\begin{aligned} {\zeta _u}^2 + \frac{u}{\zeta _u} = z_1^2, \end{aligned}$$

and which behaves as $\zeta _u(z_1) = z_1 + O(u)$ at small *u*. The solution as a series is

6.21 $$\begin{aligned} \zeta _u(z_1) = z_1 - \frac{1}{2} \sum _{k=1}^\infty \frac{(\frac{3}{2}(k-1))!}{k! \ (\frac{1}{2}(k-1))!} \ z_1^{1-3k}\ u^k. \end{aligned}$$

Its radius of convergence is

6.22 $$\begin{aligned} |u| < \frac{2 |z_1|^3 }{3\sqrt{3}} = r. \end{aligned}$$

The spectral curve ${\mathcal {S}}_u = {\mathcal {S}}+ u [z_1] = (\textrm{x}_u,\textrm{y}_u)$ is defined by the immersion

6.23 $$\begin{aligned} {\mathcal {S}}+u [z_1] : \left\{ \begin{array}{l} \textrm{x}_u(z) = z^2 + \frac{u}{\zeta _u(z_1)} \\ \textrm{y}_u(z) = \left( z + \frac{u}{2\zeta _u(z_1)(z-\zeta _u(z_1))} \right) 2z dz \end{array} \right. . \end{aligned}$$

It satisfies the equation

6.24 $$\begin{aligned} (y^2-x)(x-X_1)= u \left( y +\zeta _u(z_1)-\frac{u}{4\zeta _u^2(z_1)}\right) . \end{aligned}$$

It indeed reduces to $y^2-x=0$ when $u=0$.

It has the times

6.25 $$\begin{aligned} t_{\infty ,k}({\mathcal {S}}_u) = -2 \delta _{k,3} = t_{\infty ,k}({\mathcal {S}}) \qquad \forall \ k>0 \end{aligned}$$

and

6.26 $$\begin{aligned} t_{\zeta _u(z_1),0}({\mathcal {S}}_u)=-t_{\infty ,0}({\mathcal {S}}_u) = u. \end{aligned}$$

More generally, if $D=\sum _{i=1}^\ell \alpha '_i [z_i]$, the spectral curve ${\mathcal {S}}_u = {\mathcal {S}}+u D$ is given by:

6.27 $$\begin{aligned} {\mathcal {S}}_u={\mathcal {S}}+u D : \left\{ \begin{array}{l} \textrm{x}_u(z) = z^2 + u C_u \\ \textrm{y}_u(z) = \left( z + \frac{u}{2} \sum _{i=1}^\ell \frac{\alpha '_i}{\zeta _u(z_i)(z-\zeta _u(z_i))} \right) 2z dz \end{array} \right. \end{aligned}$$

where $C_u$ and $\zeta _u(z_i)$ for $i=1,\dots ,\ell$, are defined by the $\ell +1$ equations

6.28 $$\begin{aligned} \zeta _u(z_i)^2= &   z_i^2- u C_u, \ \ \zeta _u(z_i) = z_i +O(u) \\C_u= &   \sum _{i=1}^\ell \frac{\alpha '_i}{\zeta _u(z_i)}. \end{aligned}$$

In fact, it is easy to see that ${\mathcal {S}}+uD$ reduces to ${\mathcal {S}}$ at $u=0$ and has the times

6.29 $$\begin{aligned} t_{p,k}({\mathcal {S}}+uD) = t_{p,k}({\mathcal {S}}) + u \delta _{k,0} \sum _{i=1}^\ell \alpha '_i (\delta _{p,\zeta _u(z_i)}-\delta _{p,\infty }). \end{aligned}$$

#### Spectral curve Tau function

##### Definition 6.4

(*Spectral curve Tau function*) We want to find a function defined on the “moduli space of spectral curves” (we don’t define this moduli space here, see [36, 37, 39]), i.e., a section of a line bundle over this moduli space

6.30 $$\begin{aligned} {\mathcal {T}}({\mathcal {S}}) \end{aligned}$$

that satisfies Fay identity

6.31 $$\begin{aligned} \forall \ D=\sum _{i=1}^n [z_i]-[\tilde{z}_i] \text { supersymmetric} \quad \frac{{\mathcal {T}}({\mathcal {S}}+D)}{{\mathcal {T}}({\mathcal {S}})} = \det \frac{{\mathcal {T}}({\mathcal {S}}+[z_j]-[\tilde{z}_i])}{{\mathcal {T}}({\mathcal {S}})} . \end{aligned}$$

The solution to these Fay identities can in general not be written with classical functions (like Theta functions), they are genuinely transcendental, having the Painlevé property.

However, they can sometimes be expressed as integrals (matrix integrals), and in general they can be expressed as power series in some large times limit (also called heavy charge limit), as formal series expansion whose terms are computed by Topological Recursion, that we show in Section 6.2.

### Spectral curves Tau function

We shall describe a solution to (6.31) as an asymptotic series, thanks to Topological Recursion [33, 43].

Topological Recursion was introduced initially to compute large order asymptotic expansions of matrix integrals [21, 22, 34, 35], and was then turned into a recursive definition of algebraic invariants [17, 43],

Let $\hbar$ be a “small” parameter, it is also sometimes called the “quantum” parameter, or the “dispersion” parameter, that will scale the spectral curve. The limit $\hbar$ small will correspond to “large spectral curves” or “heavy spectral curves”.

#### Definition 6.5

(*Rescaling*) We define the rescaling by $\lambda \in {\mathbb {C}}^*$ of a spectral curve ${\mathcal {S}}={\Sigma }\hookrightarrow T^*\underline{{\Sigma }}$ with immersion map $(\textrm{x},\textrm{y})$, as the rescaling of the fiber coordinate $\textrm{y}\rightarrow \lambda \textrm{y}$:

6.32 $$\begin{aligned} \lambda {\mathcal {S}}:=({\Sigma },(\textrm{x},\lambda \textrm{y})). \end{aligned}$$

This rescales all times and periods:

6.33 $$\begin{aligned} t_{p,k}(\lambda {\mathcal {S}}) = \lambda \ t_{p,k}({\mathcal {S}}), \quad \epsilon _i(\lambda {\mathcal {S}}) = \lambda \ \epsilon _i({\mathcal {S}}). \end{aligned}$$

In particular, the spectral curve $\hbar ^{-1}{\mathcal {S}}$ has “large times” in the limit $\hbar \rightarrow 0$, and it is called a “**heavy limit**”.

The conjecture presented in [18, 38, 39, 42, 43] is that topological recursion yields a solution of Fay/Hirota (6.31), as formal series in $\hbar$:

#### Conjecture 6.1

(Tau functions from topological recursion) Let

6.34 $$\begin{aligned} \hat{\Theta }(\vec {u};\tau ) = e^{\hbar ^{-2}F_0({\mathcal {S}})+F_1({\mathcal {S}})} e^{-2\pi \textrm{i}\,(\hbar ^{-1} \epsilon -\mu ,\vec {u})} \Theta (\vec {u};\tau ) e^{-\pi \textrm{i}\,\hbar ^{-2} (\epsilon ,\tau \epsilon )} e^{2\pi \textrm{i}\,(\hbar ^{-1}\epsilon ,\chi )} e^{-2\pi \textrm{i}\,(\mu ,\chi )} e^{\pi \textrm{i}\,(\mu ,\tau \mu )}, \end{aligned}$$

where $$\chi =\nu +\tau \mu $$.

In [18, 38, 39, 42] is defined the following formal Tau function:

6.35 $$\begin{aligned} {\mathcal {T}}(\hbar ^{-1}{\mathcal {S}}) = \left( 1 + \sum _{k=1}^\infty \frac{1}{k!} \sum _{\overset{\vec m_i, \ g_i}{2g_i+|\vec m_i|-2>0}} \prod _{i=1}^k \frac{\hbar ^{2g_i+|\vec m_i|-2}}{\prod _{j=1}^{\mathfrak {g}}m_{i,j}!} F_{g_i}^{(\vec m_i)}({\mathcal {S}}) \partial ^{\vec m_i}_{\vec {u}} \right) \left. \hat{\Theta }(\vec {u};\tau ) \right| _{\vec {u}=\hbar ^{-1}\zeta ({\mathcal {S}})+\chi }, \end{aligned}$$

where $F_g({\mathcal {S}})$ and $\omega _{g,n}({\mathcal {S}})$ are the TR invariants of ${\mathcal {S}}$ (see [39, 43]), and where $\vec m_i=(m_{i,1},\dots ,m_{i,{\mathfrak {g}}})\in {\mathbb {Z}}_+^{\mathfrak {g}}$ and $|\vec m_i| = \sum _{j=1}^{\mathfrak {g}}m_{i,j}$, and

6.36 $$\begin{aligned} F_{g}^{(\vec m)}({\mathcal {S}}) = \overbrace{\oint _{\mathcal{B}_1}\dots \oint _{\mathcal{B}_1}}^{m_1} \overbrace{\oint _{\mathcal{B}_2}\dots \oint _{\mathcal{B}_2}}^{m_2} \dots \overbrace{\oint _{\mathcal{B}_{\mathfrak {g}}}\dots \oint _{\mathcal{B}_{\mathfrak {g}}}}^{m_{\mathfrak {g}}} \omega _{g,|\vec m|}({\mathcal {S}}), \end{aligned}$$

6.37 $$\begin{aligned} \partial ^{\vec m}_{\vec {u}} = \prod _{j=1}^{\mathfrak {g}}\frac{\partial ^{m_j}}{\partial u_j^{m_j}}. \end{aligned}$$

${\mathcal {T}}(\hbar ^{-1}{\mathcal {S}})$ is a formal series of $\hbar$ times an exponential $e^{\hbar ^{-2}F_0({\mathcal {S}})}$, and whose coefficients are differential polynomials of $\hat{\Theta }$. We choose a grading in $\hbar$ for which $\hat{\Theta }$ and its derivatives have degree 0. For each power of $\hbar$, there is a finite number of terms.

The 1st few terms are:

6.38 $$\begin{aligned} {\mathcal {T}}(\hbar ^{-1}{\mathcal {S}})= &   \hat{\Theta }(\hbar ^{-1}\zeta ({\mathcal {S}})+\chi ) + \hbar \Big ( \sum _{i=1}^{\mathfrak {g}}\left( \oint _{\mathcal{B}_i} \omega _{1,1}({\mathcal {S}})\right) {\hat{\Theta }}'^{(i)}\\  &   + \frac{1}{6}\sum _{i,j,k=1}^{\mathfrak {g}}\left( \oint _{\mathcal{B}_i}\oint _{\mathcal{B}_j}\oint _{\mathcal{B}_k} \omega _{0,3}({\mathcal {S}})\right) {\hat{\Theta }}'''^{(i,j,k)} \Big ) \\  &   + O(\hbar ^2). \end{aligned}$$

The conjecture is that this ${\mathcal {T}}$ satisfies Fay identities (6.31), order by order in $\hbar$.

**Proof attempts:**

- In [18], it was verified that the conjecture holds true for the first 3 orders in $\hbar$, for arbitrary compact spectral curves.
- Using Hirota/Fay vs Lax pairs. There have been proofs to all orders in $\hbar$ for certain families of spectral curves, using a Lax pair differential system, also related to the notion of quantum curve.
  Two kinds of proofs exist in the literature: either start from Topological Recursion (TR) and derive Fay identities order by order as formal series, or vice versa, start from a Tau function satisfying Fay, and proving that its formal asymptotic expansion obeys TR.
  Cases where it is proved:
  - Genus zero spectral curves are easier (there is no Theta-function term):
    - Airy curve: Bergère, Eynard 2009: “Determinantal formulae and loop equations” [10]. In this article a version of the Fay identity is proved for the case $n=2$.
    - Genus zero “topological type”: Bergère, Borot, Eynard 2013: “Rational Differential Systems, Loop Equations, and Application to the *q*th Reductions of KP” [14].
    - Genus zero for ribbon graphs: Do, Manescu, 2013: “Quantum curves for the enumeration of ribbon graphs and hypermaps” [24].
    - Genus zero Schrödinger: Mulase, Sulkowski 2015: “Spectral curves and the Schrödinger equations for the Eynard-Orantin recursion” [71].
    - Review Norbury 2016: “Quantum curves and topological recursion” [73].
    - Genus zero “topological type”: Belliard, Eynard, Marchal 2016: “Integrable differential systems of topological type and reconstruction by the topological recursion” [8], using “Loop Equations from Differential Systems on Curves” [9].
    - Genus zero for “admissible spectral curves”: Bouchard, Eynard 2016: “Reconstructing WKB from topological recursion” [19]. In this article a version of the Fay identity is proved for the case $n=2$.
    - Genus zero Hurwitz-type: Do, Dyer, Mathews 2017, “Topological recursion and a quantum curve for monotone Hurwitz numbers” [23].
    - Dunin-Barkowski, Mulase, Norbury, Popolitov, Shadrin 2017: “Quantum spectral curve for the Gromov–Witten theory of the complex projective line” [29].
    - Genus zero hypergeometric type: Bychkov, Dunin-Barkowski, Kazarian, Shadrin 2021 “Topological recursion for Kadomtsev-Petviashvili tau functions of hypergeometric type” [20]. In this article the authors relate TR to KP, which satisfies Hirota/Fay identities.
    - Genus 0: Kidwai, Osuga 2022 : “Quantum curves from refined topological recursion: the genus 0 case” [61] .
    - Weighted-Hurwitz numbers: Alexandrov, Chapuy, Eynard, Harnad, 2018 “Weighted Hurwitz numbers and topological recursion” [2–4]. In this series of articles the authors start from the Fay-Hirota property and derive the Topological Recursion as well as the quantum curve.
  - Higher genus:
    - Higher genus: Dumitrescu, Mulase 2014: “Quantum curves for Hitchin fibrations and the Eynard-Orantin theory”, “Quantization of spectral curves for meromorphic Higgs bundles through topological recursion”, [27, 28].
    - Genus 1: Iwaki, Marchal, Saenz 2016, 2017, 2018, 2020: “Quantum Curve and the First Painleve Equation” [56], “Painlevé equations, topological type property and reconstruction by the topological recursion” [55], “Painlevé equations, topological type property and reconstruction by the topological recursion” [53], “2-parameter $\tau$-function for the first Painlevé equation: topological recursion and direct monodromy problem via exact WKB analysis” [54].
    - Hyperelliptic curves: Marchal, Orantin 2022: “Quantization of hyper-elliptic curves from isomonodromic systems and topological recursion” [69], and Eynard Garcia-Failde 2023: “From topological recursion to wave functions and PDEs quantizing hyperelliptic curves” [40]. In this article a version of the Fay identity is proved for the case $n=2$.
    - Algebraic curves with only simple branch-points: Eynard, Garcia-Failde, Marchal, Orantin 2021: “Quantization of classical spectral curves via topological recursion” [44]. In this article a version of the Fay identity is proved for the case $n=2$.
  - Beyond:
    - Difference equations: Marchal 2017: “WKB solutions of difference equations and reconstruction by the topological recursion” [68].

And we might have forgotten some other proofs, in particular in cases of specific applications.

#### Remark 6.1

During the time of reviewing of this article by the journal, a quite general proof of the conjecture was obtained by Alexandrov, Bychkov, Dunin-Barkowski, Kazarian, Shadrin [5].

## Other Tau functions

We shall not be exhaustive and mention only the following example: the 1—Matrix model prototype, which is extremely useful and used with many applications from combinatorics to quantum gravity and string theory.

### Matrix integrals

Typical examples of Tau functions are matrix integrals. It is usual to consider the punctures at $\infty$ and write $x=1/\xi$, i.e., $\xi =0$ corresponds to $x=\infty$.

Let $\Gamma = {\mathbb {C}}[x]$ be the vector space of complex polynomials. To $\vec {t}\in \Gamma$ associate the polynomial, called the “potential”

7.1 $$\begin{aligned} V(x) = \sum _{k=1}^{\deg V} \frac{t_k}{k} x^k. \end{aligned}$$

Let *N* be a positive integer and $H_N$ the space of $N\times N$ Hermitian matrices, equipped with the Lebesgue measure *dM*. Let:

7.2 $$\begin{aligned} {\mathcal {T}}_N(\vec {t}) = \int _{H_N} dM e^{-{\operatorname {Tr}}V(M)}, \end{aligned}$$

where we assume that the integral is absolutely convergent (if it is not absolutely convergent on $H_N$, replace $H_N$ by $H_N(\gamma )$ the space of $N\times N$ normal matrices with eigenvalues on a Jordan arc $\gamma$, see [41]).

For a divisor $D=\sum _i \alpha _i.z_i$, the Sato shift $\vec {t}\mapsto \vec {t}+[D]$ amounts to

7.3 $$\begin{aligned} V(x) \mapsto V(x) + \sum _{i} \alpha _i \ln (x-z_i), \end{aligned}$$

(notice that the Sato-shifted potential is not a polynomial, it is a polynomial only order by order as formal series), in other words

7.4 $$\begin{aligned} {\mathcal {T}}_N(\vec {t}+[D]) = \int _{H_N} dM e^{-{\operatorname {Tr}}V(M)} \ \prod _{i=1}^\ell \det (M-z_i)^{-\alpha _i}. \end{aligned}$$

In particular with the divisor $D=[\infty ]-[x]$ we consider

7.5 $$\begin{aligned} \psi _N(\vec {t};x) = \frac{{\mathcal {T}}_N(\vec {t}-[x]+[\infty ])}{{\mathcal {T}}_N(\vec {t})} = \frac{1}{{\mathcal {T}}_N(\vec {t})}\int _{H_N} dM e^{-{\operatorname {Tr}}V(M)} \ \det (x-M), \end{aligned}$$

and with the divisor $D=[x]-[\infty ]$ we consider

7.6 $$\begin{aligned} \phi _N(\vec {t};x) = \frac{{\mathcal {T}}_N(\vec {t}+[x]-[\infty ])}{{\mathcal {T}}_N(\vec {t})} = \frac{1}{{\mathcal {T}}_N(\vec {t})}\int _{H_N} dM e^{-{\operatorname {Tr}}V(M)} \ \frac{1}{\det (x-M)}. \end{aligned}$$

It is well known in the theory of random matrices [62] that they satisfy an orthogonality relationship

7.7 $$\begin{aligned} \int _{\mathbb {R}}\psi _n(\vec {t};x) \phi _m(\vec {t};x) dx = \delta _{n,m}. \end{aligned}$$

Moreover, it is well known [1, 11–13] that for supersymmetric divisors they satisfy Hirota equations

7.8 $$\begin{aligned} \frac{{\mathcal {T}}_N(\vec {t}+\sum _{i=1}^\ell [z_i]-[\tilde{z}_i])}{{\mathcal {T}}_N(\vec {t})} = \det \frac{{\mathcal {T}}_N(\vec {t}+[z_i]-[\tilde{z}_j])}{{\mathcal {T}}_N(\vec {t})}. \end{aligned}$$

Matrix integrals do satisfy Hirota equations and Fay identities.

More generally, matrix integrals are known to be Tau functions [15, 16, 41, 48].

## Conclusion

This article presented a more modern and TR compatible reformulation of Hirota and Fay equations.

- The usual formulation of Hirota equations is with formal series, and if we multiply by the appropriate exponential factor and differential-form factor, this is formulated with a spinor trans-monomial.
- In the language of formal series or trans-monomials, Hirota equations are equivalent to Fay identities. But then Fay identities can be formulated not only as formal series, they can be extended to functions on Riemann surfaces.
- Fay identities are then naturally formulated with Riemann surfaces, and some solutions can be found in terms of Riemann surfaces.
- We recalled two solutions, one where ${\mathcal {T}}$ is defined on the space of meromorphic forms on a fixed Riemann surface, and one where ${\mathcal {T}}$ is defined on the moduli space of spectral curves, and is given asymptotically by topological recursion.
- In Topological Recursion, people often mention the “quantum curve conjecture” claiming that Tau functions Sato-shifted by unitary divisors should obey some ODE. The ODE is in fact a consequence of integrability and thus the “quantum curve conjecture” is in fact a consequence of a stronger “integrability” conjecture, i.e., the fact that the Topological-Recursion Tau function Eq. (6.35) obeys Fay identities. This conjecture has been proved in number of cases but it is still to be proved for more general cases.

## Data Availability

The authors declare that no data were used in this research.

## Footnotes

1 Alternative definitions or generalizations exist, in particular generalizations for covering maps $\textrm{x}$ of infinite degree, or with $\log$ singularities, or for non compact surfaces or non connected, etc.
